# Systematic review of the association between life-course socioeconomic status and late-life cognitive decline

**DOI:** 10.1136/jech-2025-223864

**Published:** 2025-10-22

**Authors:** Eleanor I Williams, Isla Kuhn, Carol E Brayne, Sebastian Walsh

**Affiliations:** 1 Department of Public Health and Primary Care, University of Cambridge, Cambridge, UK; 2 School of Clinical Medicine, University of Cambridge, Cambridge, UK; 3 Cambridge Public Health, University of Cambridge, Cambridge, UK

**Keywords:** DEMENTIA, Life course epidemiology, SOCIAL CLASS, Health inequalities, SYSTEMATIC REVIEW

## Abstract

**Background:**

Socioeconomic status (SES) is a potentially important upstream determinant of late-life cognitive health, but a review which captures the dynamic influence of SES across the life-course is lacking. We conducted a systematic review of studies reporting associations between life-course SES and dementia/late-life cognitive decline.

**Methods:**

On 21 February 2024, we searched Medline, Embase, PsycINFO, CINAHL, British Education Index, Web of Science, Scopus and Advanced Google for studies related to life-course SES and dementia. We included studies employing trajectory or mediation analysis that measured dementia/cognitive decline as outcomes. Two researchers independently screened articles and assessed risk of bias. Results were synthesised narratively and in Harvest plots.

**Results:**

We included 18 out of 6040 studies screened (n=7 trajectory studies, n=8 mediation studies, n=3 both). Most (13/23) trajectory analyses reported that stable low SES and downward social mobility, relative to stable high SES/upward mobility, were linked to higher dementia and/or cognitive decline risk. Half (5/10) of the mediation analyses reported full mediation of adulthood SES on the association between childhood SES and dementia/cognitive decline, and 4/10 reported partial mediation. Overall, study quality was moderate.

**Conclusion:**

SES has a dynamic life-course association with dementia risk. Increases in dementia risk are compounded by sustained life-course disadvantage. Policies to address socioeconomic disadvantage across the life-course are needed to address this upstream determinant of dementia.

**PROSPERO registration number:**

CRD42024505975.

**WHAT IS ALREADY KNOWN ON THIS TOPIC**

- Prior research, including two systematic reviews, suggests a strong association between low socioeconomic status (SES) and increased dementia risk. However, most studies have focused on cross-sectional SES measures, overlooking the influence of SES changes across different life stages.

**WHAT THIS STUDY ADDS**

- In this systematic review of 18 studies, we found that low SES throughout life was consistently associated with higher dementia risk, compared with other SES trajectories. Upward social mobility appeared to mitigate some of the risk, and adulthood SES appeared to partially mediate the association between earlier life disadvantage and dementia. Heterogeneity of exposure and outcome measures prohibited meta-analysis.

**HOW THIS STUDY MIGHT AFFECT RESEARCH, PRACTICE OR POLICY**

- This study underscores the importance of addressing SES disparities across the life-course to reduce dementia risk. Policy interventions that focus on early education and social support for children, as well as adults experiencing socioeconomic disadvantage, could help to lower dementia risk. Future studies could standardise SES measurements and look to include more diverse populations.

## Introduction

 In 2019, an estimated 55–57 million people worldwide lived with dementia, with the number expected to rise to 152.8 million by 2050 driven by increasing life expectancy.^1^ Modifiable risk factors are gaining increasing attention in dementia research, as they present opportunities for prevention and intervention, amidst the challenges of finding improved medical treatments.^2^ A 2024 Lancet Commission report found that up to 45% of dementia cases are attributable to 14 key risk factors, which occur across different life stages.^2^ Some of these modifiable risk factors include health behaviours such as smoking, physical inactivity and excess alcohol intake, while others include broader social determinants such as fewer years in education, infrequent social contact and exposure to air pollution. All of the risk factors typically cluster in areas of socioeconomic disadvantage.^2 3^

Socioeconomic status (SES) encompasses a person’s social and economic standing, including factors like education, income and occupation. This may affect a person’s late-life cognitive health directly, for example, through educational effects on the development of cognitive reserve; or indirectly, for example, by affecting the likelihood of a person engaging in certain health behaviours such as smoking, less physical activity and poorer diet.^2 4^ There is consistent literature, including two recent systematic reviews, reporting that lower individual SES is associated with greater risk of dementia and poorer late-life cognitive performance, compared with high SES.^5 6^ However, these reviews focused on studies that measured SES cross-sectionally, precluding analysis of how SES changes throughout life may impact outcomes. It is important to understand if there are critical points within the lifecourse (eg, early life) at which low SES conveys an increased dementia risk that is largely unchanged by other life experiences or whether the effect is more akin to gradual accumulation of risk across life periods, in order to inform public health policy.^7^

In this systematic review, we identified articles reporting the association between life-course SES and late-life cognitive decline (including dementia).

## Methods

The study protocol for this systematic review was pre-registered on Prospero (ID: CRD42024505975). The manuscript is written according to the Preferred Reporting Items for Systematic reviews and Meta-Analyses reporting guidelines (online supplemental materials, item 1).

### Search strategy

We developed the search strategy with a specialist medical librarian (IK). On 21 February 2024, we searched Medline and Embase via Ovid, PsycINFO, CINAHL, British Education Index, Web of Science and Scopus for terms related to ‘socioeconomic status’, ‘lifecourse/trajectory/mediation’ and ‘dementia’. Additionally, an advanced Google search was conducted on 12 March 2024 to capture grey literature. The full search strategy is available in the online supplemental materials, item 2 and was adapted from the search strategy of a recent systematic review on cross-sectional SES and dementia,^5^ with modifications to focus on life-course SES. The strategy was run in English, but we applied no limits of language or publication date.

### Inclusion/exclusion criteria and rationale

We included studies that measured the association between SES at ≥2 life-course time points against an outcome of either: (1) a valid assessment of dementia (all-cause or any common dementia subtype) or (2) the rate of cognitive decline (a change in cognitive performance measured across ≥2 time points.

Pilot searches demonstrated that the two predominant lifecourse models employed in this literature are trajectory-based analyses and mediation-based analyses. Trajectory analyses compare individuals based on their lifecourse SES pattern (eg, consistently low SES through life, compared with those with upward mobility, downward mobility or consistently high SES). This analytic design was included in order to capture whether starting (earlier life) SES is the main driver of the SES-dementia/late-life cognitive decline association or whether it is more attributable to end position (later life) SES or independent contributions from both (mobility groups significantly different from the consistent groups). Mediation analyses captured the independent association of earlier life SES on dementia/late-life cognitive decline and the degree to which later life SES explained this association.

We excluded studies where SES was measured only at the group level (eg, average educational attainment of a population, median income of an area) and where the outcome was based only on biomarkers or neuroimaging without cognitive assessment. We included any validated measure of cognitive function, including global cognitive assessments and domain-specific assessments, provided that the same measure was used at all time points.

### Screening

Deduplicated articles were uploaded to the Rayyan systematic review software. Two authors (EW and SW) independently reviewed all titles/abstracts and relevant full texts for inclusion, before meeting to resolve conflicts. Discussion with a third author was not required.

### Risk of bias assessment

The risk of bias in included studies was assessed using the Joanna Briggs Institute checklist for cohort studies (online supplemental materials, item 3), applied independently by two authors (EW and SW) with discrepancies resolved through discussion.

### Data collection process and data items

Data extraction was performed by one author (EW) using a prespecified template, which was checked for accuracy by a second author (SW). Data were collected on variables including study population, SES measures, outcome measure, statistical methods and findings. Where multiple studies used the same cohort (English Longitudinal Study of Ageing (ELSA), Health and Retirement Study (HRS), Lothian birth cohort 1936 or where the same study reported analyses in multiple cohorts, they were treated as independent analyses.

### Data synthesis

A meta-analysis was not feasible due to the heterogeneity of study designs and SES/cognitive outcome measures. Instead, the synthesis without meta-analysis (SWiM) guidance was followed (online supplemental materials, item 4). We report findings both qualitatively and in Harvest plots stratified by analytic design and outcome measure (cognition or dementia). The majority of studies combined different measures of education, occupation and/or income within their lifecourse SES assessments, meaning that we were unable to stratify syntheses by different SES constructs.

## Results

### Study selection

**Figure 1:**
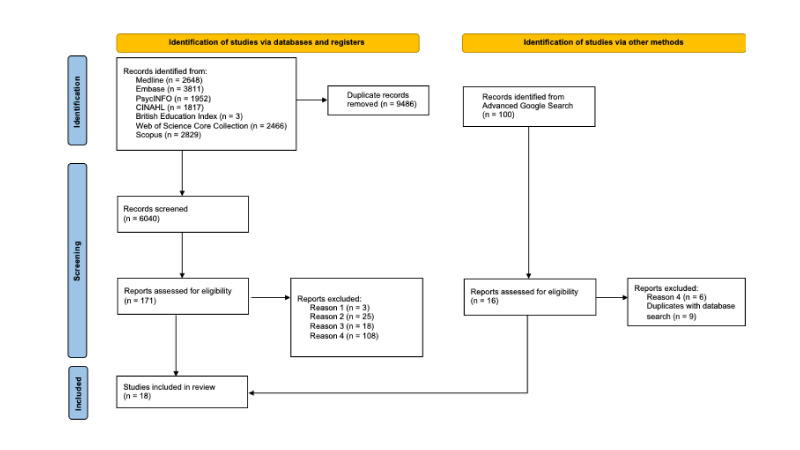
Preferred Reporting Items for Systematic Reviews and Meta-Analyses (PRISMA) flow diagram of selected studies. PRISMA flow diagram reason 1: SES measured only at the group level, reason 2: only one SES measure which is not a lifecourse measure, reason 3: cognition assessed only at a single timepoint, reason 4: methods involve analytic designs other than trajectory or mediation analysis.

We identified 6040 unique records, of which 18 met our eligibility criteria and were included^8^,^25^ (figure 1).

### Description of studies

All included studies were analyses of prospective cohort data (n=18) (online supplemental materials, item 5). Each of the 18 studies focused on older adults, with baseline ages ranging from 50 years to 70 years. Several papers drew from the same longitudinal cohorts, such as the HRS (n=5), the ELSA (n=3) and the Lothian/Aberdeen 1936 Birth Cohorts (n=3).

Most studies were conducted in high-income countries (HICs), including the UK (n=6), USA (n=7), Sweden, Switzerland, Finland and Taiwan; and one study was conducted in China. Of the 11 studies assessing cognitive decline as an outcome, the median gap between first and second cognitive assessment was 12.5 years, ranging from 2 years to 18 years.

#### Exposure

Measurement of SES varied widely across studies. All studies included a measure of early-life (childhood) SES, for example, parental education and income, household overcrowding and participants’ education level and a measure of adulthood SES, for example, income, wealth (i.e., assets) and occupational status. Most studies used composite SES indices which included combinations of variables from these domains.

#### Outcome

Of the included studies, around half (n=7) reported dementia as the outcome, with the remainder reporting cognitive decline (n=11) (online supplemental materials, item 5). Dementia status was determined using validated diagnostic criteria such as the Diagnostic and Statistical Manual of Mental Disorders or the Clinical Dementia Rating. Cognitive function was measured using a variety of validated tools, most commonly the Mini-Mental State Examination.

#### Analytic design

The 18 included studies were split between trajectory analysis (n=7), mediation analysis (n=8) and those using both (n=3). The trajectory analyses typically categorised participants into groups representing ‘stable high SES’, ‘upward mobility’, ‘downward mobility’ and ‘stable low SES’. While the mediation analyses examined how much adulthood SES mediated the association between earlier life SES measures and late-life cognitive outcomes.

### Risk of bias in studies

Overall, the included studies were at moderate risk of bias (online supplemental materials, item 6). The studies typically scored well on domains like sampling bias, validity of exposure ascertainment and the use of appropriate statistical analysis. A number of studies (n=10) insufficiently adjusted for relevant confounders. However, there is some debate about whether variables, such as smoking, alcohol and obesity, for example, are truly confounders in this context. Given the socioeconomic patterning of such factors,^3^ at least part of their effects could be considered to be on the causal pathway between SES and dementia. Some studies did adjust for these variables, and in order to adopt a conservative approach to identifying direct associations between SES and dementia, we decided to consider such factors as potential confounders that should have been adjusted for in the included studies.

### Narrative synthesis and harvest plots

**Figure 2:**
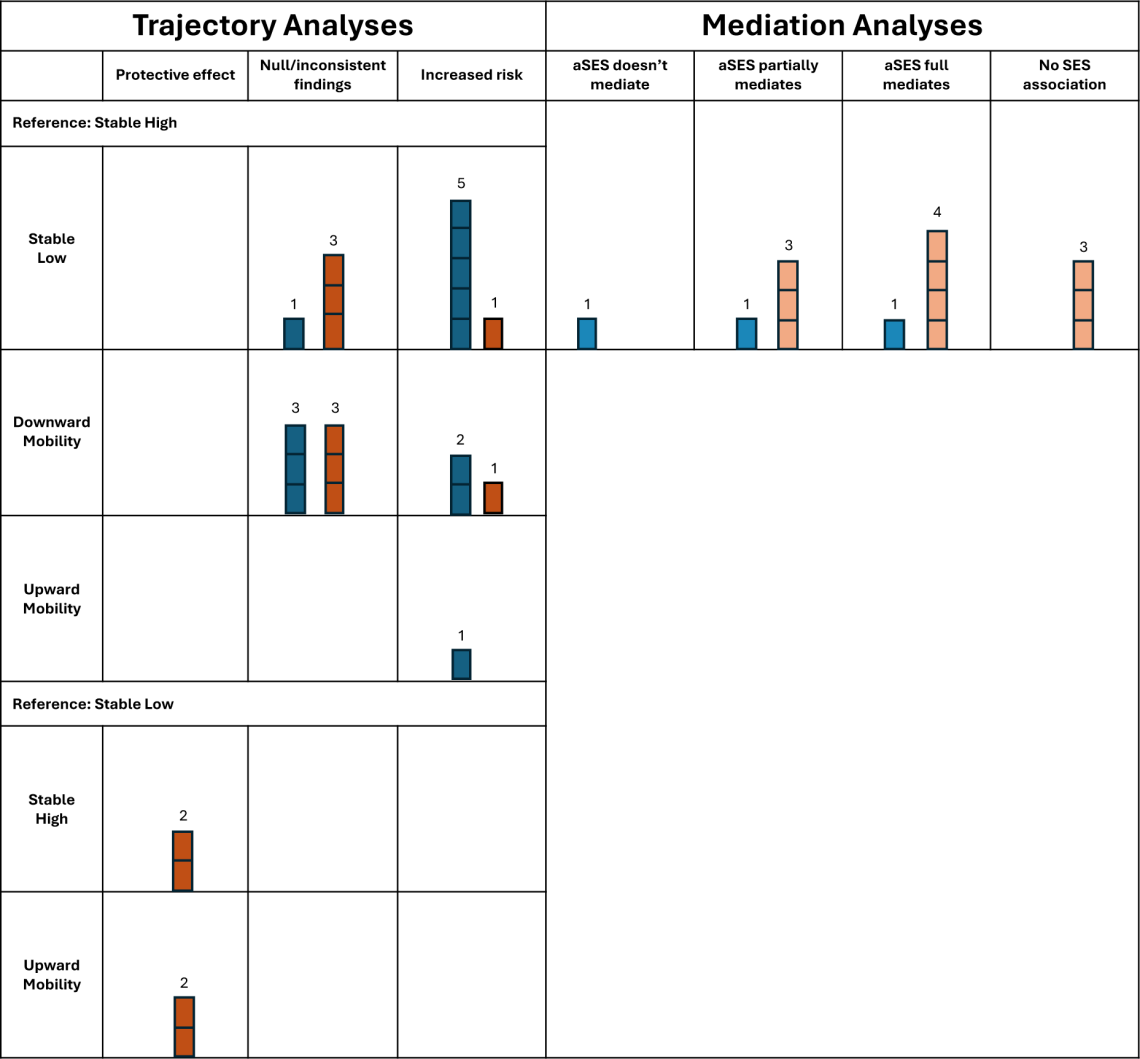
Harvest plots showing the pattern of results according to analytical design and outcome measure. Blue: studies measuring dementia as an outcome, orange: studies measuring cognitive decline as an outcome. Size of bars corresponds to number of studies with the reported finding. aSES, adulthood socioeconomic status (and reporting whether or not it fully/partially mediates the association between an earlier life (usually childhood) SES measure and the outcome).

A summary of the findings of each study and direction of effect can be found in table 1 and figure 2.

**Table 1:** Study findings and direction of effect

Author	Findings	Summary of association
Trajectory Analyses		
Cadar *et al*^19^	25% of participants developed dementia over 12 years. Low child SES increased dementia risk compared with high child SES. Education had no protective effect, but lower occupational class increased dementia risk, compared with higher occupational class categories. Lowest wealth strongly predicted dementia, independent of other SES markers. Stable-low SES across life showed higher risk compared with stable-high SES. SES mobility trends had no impact. Dementia incidence was socioeconomically patterned, mainly by wealth and long-term SES disadvantage.	Cumulative disadvantage associated with increased risk compared with stable high SES. No social mobility association
Cha *et al*^22^	Higher SES participants lived more years without dementia and experienced compressed dementia periods compared with less advantaged participants. SES across three life-course periods (child, young adult, late-life) additively affected dementia risk. Adults from disadvantaged childhoods with high education levels and wealth had dementia experiences similar to or better than those from advantaged childhoods with low education levels, highlighting the importance of cumulative exposure and that improving SES after childhood can have a compensatory effect in reducing the lifetime dementia burden.	Cumulative disadvantage associated with increased risk compared with stable high SES. Downward social mobility was associated with increased risk, compared with upward social mobility.
Marden *et al*^14^	High child SES, high school completion, college completion and high late-life income were associated with better memory function. The effect of high school completion was larger than both child SES and income. The interaction term between high school and income indicated a smaller benefit of high late-life income for those who completed high school. High school completion increased memory scores and high late-life income, compared with those with low SES at all time points. The worst SES pattern for memory function was low SES at all points, while the best was high SES at all points.	Cumulative disadvantage and downward mobility associated with increased risk of memory decline compared with stable high SES
Schrempft *et al*^11^	Individuals with life-course socioeconomic disadvantage performed worse on processing speed, verbal fluency, inhibitory control, memory and global cognitive tasks compared with those with upward social mobility. No association was found between socioeconomic conditions and cognitive flexibility, with few associations with subjective cognitive complaints. Associations were most consistent for education level. For social mobility, associations were found for verbal fluency, memory and inhibitory control in the CoLaus cohort, and for processing speed, verbal fluency and global cognitive impairment in the Vivre/Leben/Vivere cohort. Associations between socioeconomic disadvantage and cognitive decline were less consistent, suggesting socioeconomic conditions predict performance levels across cognitive domains and, to a lesser extent, performance trajectories.	Mixed results for associations between cumulative disadvantage and cognitive decline
Sindi et al	The group reporting better financial situation had a reduced risk for dementia. In contrast, the group reporting a worse financial situation did not have an increased risk for dementia. Analyses on perceptions of current financial situation showed that the groups reporting satisfaction or dissatisfaction with financial situation did not differ in risk for dementia.	Upward social mobility may reduce risk compared with stable low SES.
Staff *et al*^21^	The results show a significant decline in memory performance with age, with significant effects of practice, sex, childhood ability and child SES on memory performance. Social mobility did not significantly influence memory performance. Education, child SES, social mobility and interaction terms (Age × child SES, Age × social mobility) were not significant, but a significant age × education interaction was observed. Women performed better than men in all models. Education appears to protect memory, suggesting it supports resilience to age-related cognitive impairment. Upward social mobility did not enhance this effect, indicating resilience to age-related decline may be established in early life.	No evidence of social mobility association with cognitive decline
Zeki Al Hazzouri *et al*^9^	Dementia incidence rates were lowest among those with high SES trajectories and highest among those with low SES trajectories, followed by those with upward SES trajectories with low education and then downward SES trajectories. In fully adjusted models, continuously high SES was associated with lower hazard ratios for dementia/CIND compared with continuously low SES. In age-adjusted models, each 1-unit increase in cumulative SES disadvantage increased the hazard of dementia/CIND by 16%. Early social disadvantage may increase late-life dementia risk.	Cumulative disadvantage associated with increased risk compared with stable high SES. Upward social mobility associated with protective effect
Mediation analyses		
Baranyi *et al*^20^	Path analyses suggested that childhood neighbourhood disadvantage is indirectly linked to late-life cognitive function through lower education and selective residential mobility. Living in advantaged areas in mid-to-late adulthood may directly contribute to better cognitive function and slower decline, whereas an advantaged childhood neighbourhood likely affects functioning through cognitive reserves.	Adult SES fully mediates child SES effect on cognitive decline.
Chiao *et al*^18^	The effects of child SES on late-life cognitive decline were largely explained by adult SES, with disadvantageous adult SES exacerbating cognitive declines, when controlling for ageing, practice and other covariates. Specific components of life-course SES disadvantage found to be associated with late-life cognitive decline included household income and perceived economic strain at the study baseline.	Adult SES partially mediates child SES effect on cognitive decline.
Deckers *et al*^23^	3% of participants followed up in this study developed dementia. Higher education had no direct effect on dementia risk, but showed lower risk through its relationship with higher wealth and better lifestyle/health. These findings suggest that managing risk in those with both less wealth and low educational level might have an effect on dementia prevention by narrowing the gap in dementia risk between the rich and poor.	Adult SES fully mediates child SES effect on dementia risk.
Korhonen *et al*^24^	The largest indirect effect was observed for household crowding in childhood mediated through adult SES. The study shows that child SES is associated with dementia, and that the underlying mechanisms only partly relate to adult SES and cardiovascular health. Similar results were found for early-onset dementia.	Adult SES partially mediates child SES effect on dementia risk
Krasnova et al	High SES at each life-course stage starting in young adulthood had a protective estimated effect on global and domain-specific cognition intercepts. Compared with consistently low SES, consistently high SES and high SES beyond childhood had the largest benefit for global cognition intercepts. However, none of the life-course SES measures influenced the rate of global or domain-specific decline.	No evidence of life-course SES influencing cognitive decline
Oi and Haas^13^	High child SES leads to slower cognitive decline than low child SES, partially due to lower levels of cardiometabolic risk, which has been found to be linked to both child SES and earlier onset of cognitive impairment. However, these pathways were found to operate entirely through adult socioeconomic attainment.	Adult SES fully mediates child SES effect on cognitive decline
Racine Maurice *et al*^12^	The majority of early life SES markers were not associated with late-life cognitive decline, with the exception of maternal education. Mediation analysis of the relationship between low parental educational attainment and cognitive decline showed that the direct relationship between less educated mothers and cognitive decline was significant and the indirect relationship between less educated mothers, adult SES and cognitive decline was not significant. The indirect relationship between less educated fathers, adult SES and cognitive decline was also not significant. Testing for a possible interaction between having a highly educated mother but living in low SES conditions showed no association in relation to participants’ late life cognitive decline.	Minimal evidence for association between early life SES and cognitive decline
Zhang *et al*^8^	Among both men and women, urban residence in early life and higher levels of education were associated with lower odds of cognitive impairment at baseline. The authors found modest support for a protective effect of advantaged childhood background on the odds of cognitive impairment onset during the 2-year follow-up period, especially among women. For the oldest old men, the odds of cognitive impairment onset during the 2-year follow-up were 61% lower for participants who were most advantaged in both childhood and adulthood than for those who were disadvantaged throughout life. Among the oldest old women, the pattern was similar, where the odds of cognitive impairment onset were 53% lower for those most advantaged in both childhood and adulthood than for those with life-course disadvantage.	Adult SES partially mediates child SES effect on cognitive impairment.
Both		
Faul *et al*^17^	Trajectory: high child SES was associated with higher cognitive performance at baseline in both cohorts but did not affect the rate of cognitive decline. In both cohorts, compared with stable low SES, all other groups declined slower, with the upwardly mobile and stable high groups having the slowest decline. Mediation: respondent’s education and adult wealth fully mediated the effect of child SES on cognitive performance in the US cohort. However, in the UK cohort, child SES had a lasting effect on baseline cognitive performance partially independent of the effects of education and adult SES.	Trajectory: compared with stable low, other groups showed slower decline, with stable high and upward mobility reporting the greatest protective effects. Mediation: adult SES partially mediated child SES effect on cognitive performance in UK cohort, and fully mediated in the US cohort
Karp *et al*^16^	Trajectory: low education in combination with either low or high occupation-based SES (ie, stable low life-course SES and upward social mobility, respectively) was associated with an increased risk of Alzheimer’s disease and dementia after covariate adjustment. The combination of high education and low occupation-based SES (downward social mobility) was not associated with an increased risk of dementia/Alzheimer’s disease. Mediation: only education remained significantly associated with dementia when education and occupation-based SES were included in the same model. The association between low education and increased dementia risk when compared with high education was not mediated by adult SES or socioeconomic mobility, suggesting that early life factors may be most relevant.	Trajectory: cumulative disadvantage and upward social mobility both associated with increased risk compared with stable high SES. No downward social mobility association Mediation: no evidence that adult SES or social mobility mediates child SES association with dementia risk
Zeng *et al*^25^	Trajectory: adult SES had a stronger effect on cognitive performance than child SES, though high SES in both life stages improved late-life cognition. Accumulated SES disadvantages led to lower cognition, especially among those with life-course SES disadvantage and downward social mobility. Older adults with SES advantages tended to experience a slower cognitive decline, while those with cumulative disadvantages saw a faster decline. i’Mediation: mediation effects of adult SES on the association between child SES and cognitive outcomes were observed. Better child SES was associated with higher adult SES, which in turn was linked to improved baseline global cognition and episodic memory. Adult SES fully mediated the relationship between child SES and episodic memory trajectory. Higher SES in both child and adult life stages was linked to a faster decline in episodic memory among the total sample.	Trajectory: inconsistent pattern of results with higher SES trajectories associated with faster decline in some analyses and slower decline in others Mediation: adult SES partially mediated child SES effect on global cognition, and fully mediated the association with episodic memory

CIND, cognitive impairment not dementia; SES, socioeconomic status.

#### Trajectory studies (n=10)

Stable low SES (n=6/10), and downward mobility (n=3/6) were associated with worse late-life outcomes (either increased dementia risk or greater risk of cognitive decline), compared with stable high SES or upward mobility (table 1; figure 2). Some studies reported a dose-response relationship with a steeper upward trajectory predicting better cognitive performance compared with less upward mobility or stable low SES.^11 17^

Drawing on studies at low risk of bias, one example by Marden *et al*^14^ using data from the HRS cohort reported that stable high SES at all three measured time points (childhood, early adulthood and older adulthood) predicted the best memory function and slowest decline compared with low SES at all three time points. Completing high school had the largest estimated effect on memory function and was associated with a 22% slower rate of memory decline, compared with individuals with stable low SES. High late-life income was found to have the largest estimated benefit for slowing memory declines and was associated with a 42% slower rate of decline compared with individuals with stable low SES. The authors concluded that both early and late-life interventions may be relevant for reducing dementia risk. Other studies reported no significant association between social mobility and dementia risk or cognitive decline. One study by Karp *et al*^16^ found that regardless of upward SES trajectories, low SES at 20 years of age led to increased risk of dementia compared with stable high SES, leading the authors to conclude early life factors may be more relevant for predicting cognitive outcomes in later life. All of these studies, however, generally had smaller sample sizes and may have been underpowered.

#### Mediation studies (n=11)

The majority of these studies reported significant mediation of adulthood SES on the association between earlier life SES and dementia/cognitive impairment, with five (5/10) analyses reporting full mediation, four (4/10) reporting partial mediation, and one (1/10) reporting no evidence of mediation. Three further studies reported no association between childhood SES and the cognitive outcome, meaning any analysis for a mediating effect was irrelevant.

Korhonen *et al*^24^ (low risk of bias) reported that adulthood SES partially mediated (47%) the association between childhood household overcrowding (≥4 people per heated room) and late-life dementia risk. In contrast, there was relatively little adulthood SES mediation on the associations between single-father family structure and childhood region of residence (North/South/East/West Finland) on dementia risk (14%, 29% respectively). Another low risk of bias study, from Baranyi *et al*^20^ reported that living in a disadvantaged neighbourhood during childhood was associated with an increased likelihood of continuing to live in one in young adulthood (β=0.540; p<0.001) and mid-to-late adulthood (β=0.326; p<0.001). This pattern was associated with increased rates of cognitive decline, including steeper reductions in processing speed (β=−0.215; 95% CI −0.347 to –0.083).

#### High life-course SES and faster decline

Two studies found that high life-course SES was associated with faster cognitive decline.^22 25^ Cha *et al* also reported that high SES individuals lived more years without dementia,^22^ suggesting that these findings are examples of ‘compression of morbidity’, where increased cognitive reserve delays the onset of disease leading to a reduction in the time spent in a state of illness or disability before death.^26^

#### Findings by SES measure or by outcome

The heterogeneity of SES measures and study designs prohibited meta-analysis or meaningful disaggregation of findings between SES measures. Disaggregating studies measuring cognitive decline from those measuring dementia did not appear to change the pattern of results, though there were insufficient numbers of studies in several categories to examine this fully (figure 2). Those studies reporting global cognitive assessments, for example, the Mini Mental State Examination, generally reported protective effects of higher lifecourse SES on the rate of decline (7/8 analyses). While only 3/7 analyses measuring memory domains specifically reported significant SES associations with decline, and other domain-specific analyses (eg, processing, visuospatial domains) typically reported null associations in fully adjusted models (table 1).

#### Findings by cohort

Several studies came from analysis of the same cohort (sometimes using different waves of follow-up data). ELSA-based studies^17 19 23^ linked lower life-course SES to poorer cognitive outcomes, with some evidence that adulthood SES mediated early-life disadvantage. HRS-based studies^13 14 17 22 25^ also had similar findings that childhood and adulthood SES both shaped late-life cognition, with education and wealth offering some protective effects. LBC1936 findings were more mixed, with Baranyi *et al*^20^ reporting an indirect effect of early-life neighbourhood deprivation on cognitive outcomes via education and residential mobility, while Maurice *et al*^12^ found no significant mediating effect of adult SES on the association between child SES and cognitive decline. These findings from within the same cohorts were generally consistent with our overall pattern of results.

## Discussion

### Main findings

This systematic review, which identified 18 longitudinal studies, found a complex pattern of evidence on the associations between life-course SES and dementia/late-life cognitive decline. We found fairly consistent evidence that stable low SES throughout life was associated with higher dementia risk, compared with stable high SES groups. Evidence was more mixed for the effects of (upward and downward) social mobility and for the degree to which adulthood SES mediates the associations between earlier life SES and dementia risk/late-life cognitive decline. Overall, study quality was moderate. Low power (from either small sample sizes or limited decline among study participants during follow-up) and varying levels of confounder adjustment may explain some of the variability observed. Most data came from HICs.

Heterogeneity was observed in the methods of the included studies, particularly with regards to measures of SES. For instance, some studies assessed childhood SES based on parental factors such as parental education, income or occupation, or by measuring participants’ own education level; whereas other studies classed education in the adult or young-adult SES category. Additional child SES measures such as household overcrowding, home conditions or self-reported financial strain in childhood were also used. This methodological heterogeneity is problematic in that it limits the ability to directly compare findings across studies and prohibits meta-analysis in our review. On the other hand, SES is, by definition, a relative comparison of groups of people within specific societies^3^; therefore, a narrative synthesis has inherent value by enabling comparison of findings while respecting this cross-cultural variation.

### Findings in the context of existing literature

#### UK context

Our findings are broadly in keeping with the findings of the population-representative Medical Research Council Cognitive Function and Ageing Studies, which reported lower cognition scores (measured by MMSE) between ages 65 years and 95 years for those with lower education and from manual occupations, compared with those with higher education and working in non-manual occupations, respectively.^27^

#### Mortality and survival bias

It is established that people with higher SES tend to have longer healthy life expectancies, and longer life expectancies overall,^3^ with evidence from the UK Office for National Statistics that these inequalities have widened in recent years.^28^ This means that people from low SES backgrounds can expect to live more years of a shorter life in poor health, compared with the wealthier groups. Meanwhile, there is a consistent pattern that dementia prevalence increases exponentially with age.^29^ Therefore, any significant effects seen in the studies included in our review are in spite of a survival bias that favours people from higher SES backgrounds living longer and therefore being more likely to enter age groups where dementia is most common.

#### Education and cognitive reserve

The theory of cognitive reserve suggests that higher education preserves the brain’s ability to function in spite of neuropathology, thus delaying the onset of clinical dementia symptoms.^30^ However, once dementia manifests (at a later age), those with higher cognitive reserve experience faster cognitive decline—creating a ‘compression of morbidity’^31 32^ (though this interplay is complex and not all studies have reported this).^33^ Our findings generally align with the compression of morbidity theory, with several studies in our review finding that higher education delayed onset of dementia or cognitive impairment^14 16 22^ and that the rate of cognitive decline was more rapid in high compared with low SES groups once dementia/impairment had occurred.^22 25^

Recent studies have suggested that some of the association between education and late-life cognition/dementia risk is explained by childhood cognitive scores.^34^ Only two included studies, both analyses of long-standing Scottish birth cohorts, adjusted for childhood IQ as a potential confounder. Staff *et al* found independent contributions from early life SES, social mobility and childhood intelligence on later-life cognitive performance, but no association between social mobility and cognitive decline.^21^ Meanwhile, Baranyi *et al*^20^ found, in a model adjusted for childhood IQ score, that associations between childhood SES and cognitive decline were fully mediated through adulthood SES.

#### Recent publications since search strategy

We ran an updated search on the 20 of August 2025 (online supplemental materials, item 7) and identified five new articles (out of 1048 hits) that would have met our inclusion criteria.^35^,^39^ Sakaniwa *et al*^36^ reported that older Japanese adults with sustained high or upward SES lifecourse transitions had lower dementia risk and more dementia-free years compared with those with lower SES or downward transitions. Yu *et al*^38^ reported that those who transitioned from a low- to high-skilled job in rural South Africa appeared to have a faster late-life memory decline, compared with sustained unemployment. Which would be consistent with the education-compression of morbidity theory outlined above. Taylor *et al*^37^ reported minimal mediation of the association between lower early-life educational attainment and dementia by mid-life income in the UK Biobank cohort, with greater mediation observed via occupational complexity followed by health behaviours. Westrick *et al*^39^ reported that low wealth and gradual wealth loss through midlife was associated with poorer cognitive trajectories in the US HRS. Finally, Shi *et al*^35^ reported that upward and downward social mobility (derived from comparing participants’ education levels with that of their parents’) was associated with higher likelihood of worse cognitive trajectories, after accounting for the class origin and destination. Taken together, these articles are consistent with our overall interpretation that sustained low SES is associated with increased dementia risk/worse late-life cognitive trajectory, with evidence more mixed for the effects of social mobility and for the degree to which adulthood position mediates the risk associated with earlier-life exposure.

### Strengths and limitations

Our preregistered, double-screened systematic review has several key strengths. It is the first review to consider life-course changes in SES, rather than measuring this cross-sectionally and assuming it has been consistent across life. We employed a comprehensive search strategy across eight databases, designed with an expert medical librarian.

A key limitation of our review is the exclusion of studies measuring brain pathologies as outcomes, which restricts our understanding of the biological mechanisms linking life-course SES to cognitive decline and dementia. However, a recent systematic review examined this question and identified several of the challenges faced in this review of heterogeneous study designs and exposure/outcome variable selection.^40^

Study heterogeneity prohibited meta-analysis, and we were unable to meaningfully disentangle the potential role of different aspects of SES due to the majority of studies employing composite lifecourse SES measures. Due to a small number of studies coming from any given place, we were also unable to consider the role of context; and the life-course nature of the question means that early-life exposures relevant to cohorts born over 50 years or 75 years ago may play a different role today.

### Implications for policy, practice and research

The findings of this systematic review underscore the need for public health policies to reduce socioeconomic disparities across the life-course in order to mitigate dementia risk and support healthy late-life cognitive function. Early-life interventions, such as enhanced educational opportunities and sustained support throughout adulthood, could help build cognitive reserve and reduce midlife stressors linked to cognitive decline. Future research would benefit from more standardised SES and cognitive measures, exploring the possible mechanisms linking SES to cognitive health and diversifying geographical representation.

Amongst the included studies in this review, some researchers performed additional analyses, such as a breakdown of the SES-late life cognition association by race in the paper by Zeng *et al*,^25^ or by country of residence in the study by Faul *et al*^17^ which included a US and UK based cohort (HRS and ELSA, respectively). Zeng *et al* found that black respondents experienced more pronounced effects of cumulative SES disadvantage on cognitive decline compared with white respondents, with adulthood SES playing a stronger role in maintaining mental status among black compared with white participants. Increasing interest is being paid to ‘intersectionality’,^25^ understanding that disadvantage tends to cluster, and that it may have an additive or even multiplicative effect for those who experience it across several domains (eg, SES, gender, sexuality and ethnicity). More detailed scrutiny of these important questions is warranted, and it is important that epidemiological studies collect relevant data on these characteristics to enable such analyses. Faul *et al* highlighted notable cross-national differences, with SES mobility having more protective cognitive effects in the UK compared with the USA, where lower baseline SES and greater SES disparities appeared to exacerbate cognitive decline. While these studies provide valuable perspectives, their unique focus limits direct comparison with the broader set of studies in this review. Addressing these areas in future research could deepen understanding of the complex interactions between SES, demographic contexts and cognitive outcomes.

## Conclusion

We found evidence that SES has a dynamic life-course association with dementia risk. Increases in dementia risk are compounded by sustained life-course disadvantage. Policies to address socioeconomic disadvantage across the life-course, including both earlier and mid-life stages, are needed to address this important, upstream determinant of dementia.

## Supplementary material

10.1136/jech-2025-223864 online supplemental file 1

## Data Availability

Data are available in a public, open access repository.
