# Supplementary material for: Systematic review of the association between life-course socioeconomic status and late-life cognitive decline
Source: J Epidemiol Community Health. 2025 Oct 22;80(2):e223864. doi: 10.1136/jech-2025-223864 (PMC12911656; doi:10.1136/jech-2025-223864)
Supplement: online supplemental file 1 [file jech-80-2-s001.docx]

# Supplementary Materials

Item 1 – PRISMA checklist

Item 2 – Full Search Strategy 21^st^ February 2024

Item 3 - JBI Critical Appraisal Checklist for Cohort Studies

Item 4 – SWiM Checklist

Item 6 – Data extraction table

Item 7 – Risk of bias assessment table

Item 8 – Updated search 20^th^ August 2025

## Item 1 – PRISMA checklist

| **Section and Topic** | **Item #** | **Checklist item** | **Location where item is reported** |
| --- | --- | --- | --- |
| **TITLE** | | |  |
| Title | 1 | Identify the report as a systematic review. | Title (p.1) |
| **ABSTRACT** | | |  |
| Abstract | 2 | See the PRISMA 2020 for Abstracts checklist. | Abstract (p.2) |
| **INTRODUCTION** | | |  |
| Rationale | 3 | Describe the rationale for the review in the context of existing knowledge. | Introduction (p.4) |
| Objectives | 4 | Provide an explicit statement of the objective(s) or question(s) the review addresses. | Introduction (p.4) |
| **METHODS** | | |  |
| Eligibility criteria | 5 | Specify the inclusion and exclusion criteria for the review and how studies were grouped for the syntheses. | Methods (p.5) |
| Information sources | 6 | Specify all databases, registers, websites, organisations, reference lists and other sources searched or consulted to identify studies. Specify the date when each source was last searched or consulted. | Methods (p.4-5) |
| Search strategy | 7 | Present the full search strategies for all databases, registers and websites, including any filters and limits used. | Methods (p.4-5) |
| Selection process | 8 | Specify the methods used to decide whether a study met the inclusion criteria of the review, including how many reviewers screened each record and each report retrieved, whether they worked independently, and if applicable, details of automation tools used in the process. | Methods (p.5) |
| Data collection process | 9 | Specify the methods used to collect data from reports, including how many reviewers collected data from each report, whether they worked independently, any processes for obtaining or confirming data from study investigators, and if applicable, details of automation tools used in the process. | Methods (p.5) |
| Data items | 10a | List and define all outcomes for which data were sought. Specify whether all results that were compatible with each outcome domain in each study were sought (e.g. for all measures, time points, analyses), and if not, the methods used to decide which results to collect. | Methods (p.5) |
|  | 10b | List and define all other variables for which data were sought (e.g. participant and intervention characteristics, funding sources). Describe any assumptions made about any missing or unclear information. | Methods (p.5) |
| Study risk of bias assessment | 11 | Specify the methods used to assess risk of bias in the included studies, including details of the tool(s) used, how many reviewers assessed each study and whether they worked independently, and if applicable, details of automation tools used in the process. | Methods (p.5) |
| Effect measures | 12 | Specify for each outcome the effect measure(s) (e.g. risk ratio, mean difference) used in the synthesis or presentation of results. | Methods (p.5) |
| Synthesis methods | 13a | Describe the processes used to decide which studies were eligible for each synthesis (e.g. tabulating the study intervention characteristics and comparing against the planned groups for each synthesis (item #5)). | Methods (p.5) |
|  | 13b | Describe any methods required to prepare the data for presentation or synthesis, such as handling of missing summary statistics, or data conversions. | N/A |
|  | 13c | Describe any methods used to tabulate or visually display results of individual studies and syntheses. | Methods |
|  | 13d | Describe any methods used to synthesize results and provide a rationale for the choice(s). If meta-analysis was performed, describe the model(s), method(s) to identify the presence and extent of statistical heterogeneity, and software package(s) used. | Methods (p.5) |
|  | 13e | Describe any methods used to explore possible causes of heterogeneity among study results (e.g. subgroup analysis, meta-regression). | N/A |
|  | 13f | Describe any sensitivity analyses conducted to assess robustness of the synthesized results. | N/A |
| Reporting bias assessment | 14 | Describe any methods used to assess risk of bias due to missing results in a synthesis (arising from reporting biases). | N/A |
| Certainty assessment | 15 | Describe any methods used to assess certainty (or confidence) in the body of evidence for an outcome. | N/A |
| **RESULTS** | | |  |
| Study selection | 16a | Describe the results of the search and selection process, from the number of records identified in the search to the number of studies included in the review, ideally using a flow diagram. | Results (p.5, p.25) |
|  | 16b | Cite studies that might appear to meet the inclusion criteria, but which were excluded, and explain why they were excluded. | N/A |
| Study characteristics | 17 | Cite each included study and present its characteristics. | Results (p.6) and Table 1 (p.15-20) |
| Risk of bias in studies | 18 | Present assessments of risk of bias for each included study. | Results (p.6) and Table 2 (p.21) |
| Results of individual studies | 19 | For all outcomes, present, for each study: (a) summary statistics for each group (where appropriate) and (b) an effect estimate and its precision (e.g. confidence/credible interval), ideally using structured tables or plots. | N/A |
| Results of syntheses | 20a | For each synthesis, briefly summarise the characteristics and risk of bias among contributing studies. | Results (p.6-8) |
|  | 20b | Present results of all statistical syntheses conducted. If meta-analysis was done, present for each the summary estimate and its precision (e.g. confidence/credible interval) and measures of statistical heterogeneity. If comparing groups, describe the direction of the effect. | Results |
|  | 20c | Present results of all investigations of possible causes of heterogeneity among study results. | N/A |
|  | 20d | Present results of all sensitivity analyses conducted to assess the robustness of the synthesized results. | N/A |
| Reporting biases | 21 | Present assessments of risk of bias due to missing results (arising from reporting biases) for each synthesis assessed. | N/A |
| Certainty of evidence | 22 | Present assessments of certainty (or confidence) in the body of evidence for each outcome assessed. | Results (p.7-8) |
| **DISCUSSION** | | |  |
| Discussion | 23a | Provide a general interpretation of the results in the context of other evidence. | Discussion (p.9-10) |
|  | 23b | Discuss any limitations of the evidence included in the review. | Discussion (p.9) |
|  | 23c | Discuss any limitations of the review processes used. | Discussion (p.10) |
|  | 23d | Discuss implications of the results for practice, policy, and future research. | Discussion (p.10-11) |
| **OTHER INFORMATION** | | |  |
| Registration and protocol | 24a | Provide registration information for the review, including register name and registration number, or state that the review was not registered. | Methods (p.4) |
|  | 24b | Indicate where the review protocol can be accessed, or state that a protocol was not prepared. | Methods (p.4) |
|  | 24c | Describe and explain any amendments to information provided at registration or in the protocol. | N/A |
| Support | 25 | Describe sources of financial or non-financial support for the review, and the role of the funders or sponsors in the review. | Title Page (p.1) |
| Competing interests | 26 | Declare any competing interests of review authors. | Title Page (p.1) |
| Availability of data, code and other materials | 27 | Report which of the following are publicly available and where they can be found: template data collection forms; data extracted from included studies; data used for all analyses; analytic code; any other materials used in the review. | Title Page (p.1) |

*From:*  Page MJ, McKenzie JE, Bossuyt PM, Boutron I, Hoffmann TC, Mulrow CD, et al. The PRISMA 2020 statement: an updated guideline for reporting systematic reviews. BMJ 2021;372:n71. doi: 10.1136/bmj.n71. This work is licensed under CC BY 4.0. To view a copy of this license, visit <https://creativecommons.org/licenses/by/4.0/>

## Item 2 – Full Search Strategy

Searches were run on 21 February 2024. There were no limits of language or publication date and all databases were run from inception.

**Medline**

Ovid MEDLINE(R) and Epub Ahead of Print, In-Process, In-Data-Review & Other Non-Indexed Citations, Daily and Versions <1946 to February 21, 2024>

1. (Social class or Social position or Social standing or Social status or socioeconomic or Socio*economic status or Socio*economic class or Socio*economic position or Socio*economic standing or education or occupation or income or poverty or wealth).ti,ab,kw,kf. or exp Social Class/ or Socioeconomic Factors/ or Sociodemographic Factors/ or income/ or exp career mobility/ or economic status/ or educational status/ 1103445
2. (Dementia or Alzheimer* Disease or Vascular dementia or Lewy Bod* or mixed dementia or Cognitive decline or Cognitive reserve or Cognitive resilience or Brain reserve or Cognitive Impairment).ti,ab,kw,kf. or Dementia/ or Alzheimer Disease/ or Dementia, Vascular/ or Lewy Bod*/ or Mixed Dementias/ 363644
3. (longitudinal stud* or longitudinal survey* or longitudinal analys* or prospective stud* or prospective cohort* or follow up or followup* or follow-up stud* or cohort stud* or mediation analys* or association analys* or attenuat* or inciden* or prevalen* or lifecourse or life course or life-course).ti,ab,kw,kf. or longitudinal studies/ or prospective studies/ or Cohort Studies/ 4421109
4. (trajectory or life course or lifecourse or mediat* or attenuat* or trend* or change or childhood or adulthood).ti,ab,kw,kf. or Social Mobility/ or exp career mobility/ 4093447
5. 1 and 2 and 3 and 4 2648

**Embase**

Embase <1974 to 2024 February 20>

1 (Social class or Social position or Social standing or Social status or socioeconomic or Socio*economic status or Socio*economic class or Socio*economic position or Socio*economic standing or education or occupation or income or poverty or wealth).ti,ab. 1142569

2 exp *social class/ or exp *socioeconomics/ or exp *sociodemographics/ or exp *income/ or exp *career mobility/ or exp *economic status/ or exp *educational status/ 280193

3 1 or 2 1343172

4 (Dementia or Alzheimer* Disease or Vascular dementia or Lewy Bod* or mixed dementia or Cognitive decline or Cognitive reserve or Cognitive resilience or Brain reserve or Cognitive Impairment).ti,ab. 462928

5 exp *dementia/ or exp *Alzheimer disease/ or exp *multiinfarct dementia/ or exp *Lewy body/ or exp *mixed dementia/ 271762

6 4 or 5 521731

7 (longitudinal stud* or longitudinal survey* or longitudinal analys* or prospective stud* or prospective cohort* or follow up or followup* or follow-up stud* or cohort stud* or mediation analys* or association analys* or attenuat* or inciden* or prevalen* or lifecourse or life course or life-course).ti,ab. 5654078

8 exp *longitudinal study/ or exp *prospective study/ or exp *cohort analysis/ 95260

9 7 or 8 5670433

10 (trajectory or life course or lifecourse or mediat* or attenuat* or trend* or change or childhood or adulthood).ti,ab. 5178991

11 exp *social mobility/ or exp *career mobility/ 5338

12 10 or 11 5183959

13 3 and 6 and 9 and 12 3811

**Web of Science Core Collection**

1: ( TI= ( ( "longitudinal stud*" OR "longitudinal survey*" OR "longitudinal analys*" OR "prospective stud*" OR "prospective cohort*" OR "follow up" OR followup* OR "follow-up stud*" OR "cohort stud*" OR "mediation analys*" OR "association analys*" OR attenuat* OR inciden* OR prevalen* OR lifecourse OR "life course" OR "life-course" ) ) ) or ( AB= ( ( "longitudinal stud*" OR "longitudinal survey*" OR "longitudinal analys*" OR "prospective stud*" OR "prospective cohort*" OR "follow up" OR followup* OR "follow-up stud*" OR "cohort stud*" OR "mediation analys*" OR "association analys*" OR attenuat* OR inciden* OR prevalen* OR lifecourse OR "life course" OR "life-course" ) ) )

Results: 4225185

2: ( TI= ( ( dementia OR "alzheimer* disease" OR "vascular dementia" OR "lewy bod*" OR "mixed dementia" OR "cognitive decline" OR "cognitive reserve" OR "cognitive resilience" OR "brain reserve" OR "cognitive impairment" ) ) ) or ( AB= ( ( dementia OR "alzheimer* disease" OR "vascular dementia" OR "lewy bod*" OR "mixed dementia" OR "cognitive decline" OR "cognitive reserve" OR "cognitive resilience" OR "brain reserve" OR "cognitive impairment" ) ) )

Results: 264344

3: ( TI= ( ( "social class" OR "social position" OR "social standing" OR "social status" OR socioeconomic OR "socio?economic status" OR "socio?economic class" OR "socio?economic position" OR "socio?economic standing" OR education OR occupation OR income OR poverty OR wealth ) ) ) OR ( AB= ( ( "social class" OR "social position" OR "social standing" OR "social status" OR socioeconomic OR "socio?economic status" OR "socio?economic class" OR "socio?economic position" OR "socio?economic standing" OR education OR occupation OR income OR poverty OR wealth ) ) )

Results: 1768592

4: ( TI= ( ( trajectory OR "life course" OR lifecourse OR mediat* OR attenuat* OR trend* OR change OR childhood OR adulthood ) ) ) OR ( AB= ( ( trajectory OR "life course" OR lifecourse OR mediat* OR attenuat* OR trend* OR change OR childhood OR adulthood ) ) )

Results: 10107834

5: #1 AND #2 AND #3 AND #4

Results: 2466

**Scopus**

( TITLE-ABS ( ( "longitudinal stud*" OR "longitudinal survey*" OR "longitudinal analys*" OR "prospective stud*" OR "prospective cohort*" OR "follow up" OR followup* OR "follow-up stud*" OR "cohort stud*" OR "mediation analys*" OR "association analys*" OR attenuat* OR inciden* OR prevalen* OR lifecourse OR "life course" OR "life-course" ) ) ) AND ( TITLE-ABS ( ( dementia OR "alzheimer* disease" OR "vascular dementia" OR "lewy bod*" OR "mixed dementia" OR "cognitive decline" OR "cognitive reserve" OR "cognitive resilience" OR "brain reserve" OR "cognitive impairment" ) ) ) AND ( TITLE-ABS ( ( "social class" OR "social position" OR "social standing" OR "social status" OR socioeconomic OR "socio?economic status" OR "socio?economic class" OR "socio?economic position" OR "socio?economic standing" OR education OR occupation OR income OR poverty OR wealth ) ) ) AND ( TITLE-ABS ( ( trajectory OR "life course" OR lifecourse OR mediat* OR attenuat* OR trend* OR change OR childhood OR adulthood ) ) )

**CINAHL**

| **#** | **Query** | **Limiters/Expanders** | **Last Run Via** | **Results** |
| --- | --- | --- | --- | --- |
| S1 | TI ( (Social class or Social position or Social standing or Social status or socioeconomic or Socio*economic status or Socio*economic class or Socio*economic position or Socio*economic standing or education or occupation or income or poverty or wealth) ) OR AB ( (Social class or Social position or Social standing or Social status or socioeconomic or Socio*economic status or Socio*economic class or Socio*economic position or Socio*economic standing or education or occupation or income or poverty or wealth)) | Expanders - Apply equivalent subjects  Search modes - Boolean/Phrase | Interface - EBSCOhost Research Databases  Search Screen - Advanced Search  Database - CINAHL | 445,703 |
| S2 | (MH "Social Class+") OR (MH "Social Status") OR (MH "Socioeconomic Factors+") OR (MH "Sociodemographic Factors") OR (MH "Income+") OR (MH "Career Mobility+") OR (MH "Economic Status") OR (MH "Educational Status") | Expanders - Apply equivalent subjects  Search modes - Boolean/Phrase | Interface - EBSCOhost Research Databases  Search Screen - Advanced Search  Database - CINAHL | 415,439 |
| S3 | S1 OR S2 | Expanders - Apply equivalent subjects  Search modes - Boolean/Phrase | Interface - EBSCOhost Research Databases  Search Screen - Advanced Search  Database - CINAHL | 746,987 |
| S4 | TI ( (Dementia or Alzheimer* Disease or Vascular dementia or Lewy Bod* or mixed dementia or Cognitive decline or Cognitive reserve or Cognitive resilience or Brain reserve or Cognitive Impairment) ) OR AB ( (Dementia or Alzheimer* Disease or Vascular dementia or Lewy Bod* or mixed dementia or Cognitive decline or Cognitive reserve or Cognitive resilience or Brain reserve or Cognitive Impairment) ) | Expanders - Apply equivalent subjects  Search modes - Boolean/Phrase | Interface - EBSCOhost Research Databases  Search Screen - Advanced Search  Database - CINAHL | 112,491 |
| S5 | (MH "Dementia+") OR (MH "Dementia, Vascular+") OR (MH "Lewy Body Disease") OR (MH "Alzheimer's Disease") OR (MH "Mixed Dementias") | Expanders - Apply equivalent subjects  Search modes - Boolean/Phrase | Interface - EBSCOhost Research Databases  Search Screen - Advanced Search  Database - CINAHL | 85,111 |
| S6 | S4 OR S5 | Expanders - Apply equivalent subjects  Search modes - Boolean/Phrase | Interface - EBSCOhost Research Databases  Search Screen - Advanced Search  Database - CINAHL | 134,243 |
| S7 | TI ( (longitudinal stud* or longitudinal survey* or longitudinal analys* or prospective stud* or prospective cohort* or follow up or followup* or follow-up stud* or cohort stud* or mediation analys* or association analys* or attenuat* or inciden* or prevalen* or lifecourse or life course or life-course) ) OR AB ( (longitudinal stud* or longitudinal survey* or longitudinal analys* or prospective stud* or prospective cohort* or follow up or followup* or follow-up stud* or cohort stud* or mediation analys* or association analys* or attenuat* or inciden* or prevalen* or lifecourse or life course or life-course) ) | Expanders - Apply equivalent subjects  Search modes - Boolean/Phrase | Interface - EBSCOhost Research Databases  Search Screen - Advanced Search  Database - CINAHL | 978,925 |
| S8 | (MH "Prospective Studies+") | Expanders - Apply equivalent subjects  Search modes - Boolean/Phrase | Interface - EBSCOhost Research Databases  Search Screen - Advanced Search  Database - CINAHL | 533,370 |
| S9 | S7 OR S8 | Expanders - Apply equivalent subjects  Search modes - Boolean/Phrase | Interface - EBSCOhost Research Databases  Search Screen - Advanced Search  Database - CINAHL | 1,243,372 |
| S10 | TI ( (trajectory or life course or lifecourse or mediat* or attenuat* or trend* or change or childhood or adulthood) ) OR AB ( (trajectory or life course or lifecourse or mediat* or attenuat* or trend* or change or childhood or adulthood) ) | Expanders - Apply equivalent subjects  Search modes - Boolean/Phrase | Interface - EBSCOhost Research Databases  Search Screen - Advanced Search  Database - CINAHL | 951,573 |
| S11 | (MH "Social Mobility") OR (MH "Career Mobility+") | Expanders - Apply equivalent subjects  Search modes - Boolean/Phrase | Interface - EBSCOhost Research Databases  Search Screen - Advanced Search  Database - CINAHL | 8,576 |
| S12 | S10 OR S11 | Expanders - Apply equivalent subjects  Search modes - Boolean/Phrase | Interface - EBSCOhost Research Databases  Search Screen - Advanced Search  Database - CINAHL | 959,046 |
| S13 | S3 AND S6 AND S9 AND S12 | Expanders - Apply equivalent subjects  Search modes - Boolean/Phrase | Interface - EBSCOhost Research Databases  Search Screen - Advanced Search  Database - CINAHL | 1,817 |

**British Education Index**

| **#** | **Query** | **Limiters/Expanders** | **Last Run Via** | **Results** |
| --- | --- | --- | --- | --- |
| S5 | S1 AND S2 AND S3 AND S4 | Expanders - Apply equivalent subjects Search modes - Boolean/Phrase | Interface - EBSCOhost Research Databases Search Screen - Basic Search Database - British Education Index | 3 |
| S4 | TI ( (trajectory or life course or lifecourse or mediat* or attenuat* or trend* or change or childhood or adulthood) ) OR AB ( (trajectory or life course or lifecourse or mediat* or attenuat* or trend* or change or childhood or adulthood) ) | Expanders - Apply equivalent subjects Search modes - Boolean/Phrase | Interface - EBSCOhost Research Databases Search Screen - Basic Search Database - British Education Index | 35,588 |
| S3 | TI ( (longitudinal stud* or longitudinal survey* or longitudinal analys* or prospective stud* or prospective cohort* or follow up or followup* or follow-up stud* or cohort stud* or mediation analys* or association analys* or attenuat* or inciden* or prevalen* or lifecourse or life course or life-course) ) OR AB ( (longitudinal stud* or longitudinal survey* or longitudinal analys* or prospective stud* or prospective cohort* or follow up or followup* or follow-up stud* or cohort stud* or mediation analys* or association analys* or attenuat* or inciden* or prevalen* or lifecourse or life course or life-course) ) | Expanders - Apply equivalent subjects Search modes - Boolean/Phrase | Interface - EBSCOhost Research Databases Search Screen - Basic Search Database - British Education Index | 10,165 |
| S2 | TI ( (Dementia or Alzheimer* Disease or Vascular dementia or Lewy Bod* or mixed dementia or Cognitive decline or Cognitive reserve or Cognitive resilience or Brain reserve or Cognitive Impairment) ) OR AB ( (Dementia or Alzheimer* Disease or Vascular dementia or Lewy Bod* or mixed dementia or Cognitive decline or Cognitive reserve or Cognitive resilience or Brain reserve or Cognitive Impairment) ) | Expanders - Apply equivalent subjects Search modes - Boolean/Phrase | Interface - EBSCOhost Research Databases Search Screen - Basic Search Database - British Education Index | 397 |
| S1 | TI ( (Social class or Social position or Social standing or Social status or socioeconomic or Socio*economic status or Socio*economic class or Socio*economic position or Socio*economic standing or education or occupation or income or poverty or wealth) ) OR AB ( (Social class or Social position or Social standing or Social status or socioeconomic or Socio*economic status or Socio*economic class or Socio*economic position or Socio*economic standing or education or occupation or income or poverty or wealth)) | Expanders - Apply equivalent subjects Search modes - Boolean/Phrase | Interface - EBSCOhost Research Databases Search Screen - Basic Search Database - British Education Index | 108,243 |

**PsycINFO**

| **#** | **Query** | **Limiters/Expanders** | **Last Run Via** | **Results** |
| --- | --- | --- | --- | --- |
| S13 | S3 AND S6 AND S9 AND S12 | Expanders - Apply equivalent subjects Search modes - Boolean/Phrase | Interface - EBSCOhost Research Databases Search Screen - Advanced Search Database - APA PsycInfo | 1,952 |
| S12 | S10 OR S11 | Expanders - Apply equivalent subjects Search modes - Boolean/Phrase | Interface - EBSCOhost Research Databases Search Screen - Advanced Search Database - APA PsycInfo | 1,187,852 |
| S11 | DE "Social Mobility" | Expanders - Apply equivalent subjects Search modes - Boolean/Phrase | Interface - EBSCOhost Research Databases Search Screen - Advanced Search Database - APA PsycInfo | 2,131 |
| S10 | TI ( (trajectory or life course or lifecourse or mediat* or attenuat* or trend* or change or childhood or adulthood) ) OR AB ( (trajectory or life course or lifecourse or mediat* or attenuat* or trend* or change or childhood or adulthood) ) | Expanders - Apply equivalent subjects Search modes - Boolean/Phrase | Interface - EBSCOhost Research Databases Search Screen - Advanced Search Database - APA PsycInfo | 1,186,427 |
| S9 | S7 OR S8 | Expanders - Apply equivalent subjects Search modes - Boolean/Phrase | Interface - EBSCOhost Research Databases Search Screen - Advanced Search Database - APA PsycInfo | 629,874 |
| S8 | ((DE "Longitudinal Studies" OR DE "Prospective Studies")) OR (DE "Cohort Analysis") | Expanders - Apply equivalent subjects Search modes - Boolean/Phrase | Interface - EBSCOhost Research Databases Search Screen - Advanced Search Database - APA PsycInfo | 88,202 |
| S7 | TI ( (longitudinal stud* or longitudinal survey* or longitudinal analys* or prospective stud* or prospective cohort* or follow up or followup* or follow-up stud* or cohort stud* or mediation analys* or association analys* or attenuat* or inciden* or prevalen* or lifecourse or life course or life-course) ) OR AB ( (longitudinal stud* or longitudinal survey* or longitudinal analys* or prospective stud* or prospective cohort* or follow up or followup* or follow-up stud* or cohort stud* or mediation analys* or association analys* or attenuat* or inciden* or prevalen* or lifecourse or life course or life-course) ) | Expanders - Apply equivalent subjects Search modes - Boolean/Phrase | Interface - EBSCOhost Research Databases Search Screen - Advanced Search Database - APA PsycInfo | 597,385 |
| S6 | S4 OR S5 | Expanders - Apply equivalent subjects Search modes - Boolean/Phrase | Interface - EBSCOhost Research Databases Search Screen - Advanced Search Database - APA PsycInfo | 162,664 |
| S5 | DE "Dementia" OR DE "AIDS Dementia Complex" OR DE "Alzheimer's Disease" OR DE "Dementia with Lewy Bodies" OR DE "Frontotemporal Lobar Degeneration" OR DE "Presenile Dementia" OR DE "Pseudodementia" OR DE "Senile Dementia" OR DE "Vascular Dementia" | Expanders - Apply equivalent subjects Search modes - Boolean/Phrase | Interface - EBSCOhost Research Databases Search Screen - Advanced Search Database - APA PsycInfo | 97,536 |
| S4 | TI ( (Dementia or Alzheimer* Disease or Vascular dementia or Lewy Bod* or mixed dementia or Cognitive decline or Cognitive reserve or Cognitive resilience or Brain reserve or Cognitive Impairment) ) OR AB ( (Dementia or Alzheimer* Disease or Vascular dementia or Lewy Bod* or mixed dementia or Cognitive decline or Cognitive reserve or Cognitive resilience or Brain reserve or Cognitive Impairment) ) | Expanders - Apply equivalent subjects Search modes - Boolean/Phrase | Interface - EBSCOhost Research Databases Search Screen - Advanced Search Database - APA PsycInfo | 157,402 |
| S3 | S1 OR S2 | Expanders - Apply equivalent subjects Search modes - Boolean/Phrase | Interface - EBSCOhost Research Databases Search Screen - Basic Search Database - APA PsycInfo | 629,258 |
| S2 | ((DE "Social Class" OR DE "Disadvantaged" OR DE "Lower Socioeconomic Status" OR DE "Middle Socioeconomic Status" OR DE "Upper Socioeconomic Status") OR (DE "Socioeconomic Factors" OR DE "Economic Disadvantage" OR DE "Economic Resources" OR DE "Employment Status" OR DE "Income Level" OR DE "Social Class" OR DE "Social Disadvantage" OR DE "Socioeconomic Disparities" OR DE "Socioeconomic Status")) OR (DE "Income Level" OR DE "Lower Income Level" OR DE "Middle Income Level" OR DE "Upper Income Level") | Expanders - Apply equivalent subjects Search modes - Boolean/Phrase | Interface - EBSCOhost Research Databases Search Screen - Advanced Search Database - APA PsycInfo | 121,197 |
| S1 | TI ( (Social class or Social position or Social standing or Social status or socioeconomic or Socio*economic status or Socio*economic class or Socio*economic position or Socio*economic standing or education or occupation or income or poverty or wealth) ) OR AB ( (Social class or Social position or Social standing or Social status or socioeconomic or Socio*economic status or Socio*economic class or Socio*economic position or Socio*economic standing or education or occupation or income or poverty or wealth)) | Expanders - Apply equivalent subjects Search modes - Boolean/Phrase | Interface - EBSCOhost Research Databases Search Screen - Advanced Search Database - APA PsycInfo | 576,925 |

## Item 3 – JBI Critical Appraisal Checklist for cohort studies

A detailed explanation of all checklist options can be found under ‘cohort studies’ at: <https://jbi.global/critical-appraisal-tools>

Reviewer ______________________________________ Date_______________________________

Author_______________________________________ Year_________ Record Number_________

|  | Yes | No | Unclear | Not applicable |
| --- | --- | --- | --- | --- |
| 1. Were the two groups similar and recruited from the same population? | □ | □ | □ | □ |
| 1. Were the exposures measured similarly to assign people to both exposed and unexposed groups? | □ | □ | □ | □ |
| 1. Was the exposure measured in a valid and reliable way? | □ | □ | □ | □ |
| 1. Were confounding factors identified? | □ | □ | □ | □ |
| 1. Were strategies to deal with confounding factors stated? | □ | □ | □ | □ |
| 1. Were the groups/participants free of the outcome at the start of the study (or at the moment of exposure)? | □ | □ | □ | □ |
| 1. Were the outcomes measured in a valid and reliable way? | □ | □ | □ | □ |
| 1. Was the follow up time reported and sufficient to be long enough for outcomes to occur? | □ | □ | □ | □ |
| 1. Was follow up complete, and if not, were the reasons to loss to follow up described and explored? | □ | □ | □ | □ |
| 1. Were strategies to address incomplete follow up utilized? | □ | □ | □ | □ |
| 1. Was appropriate statistical analysis used? | □ | □ | □ | □ |

Overall appraisal: Include □ Exclude □ Seek further info □

Comments (Including reason for exclusion)

## Item 4 – SWiM Checklist

| **SWiM is intended to complement and be used as an extension to PRISMA** | | | |
| --- | --- | --- | --- |
| **SWiM reporting item** | **Item description** | **Page in manuscript where item is reported** | **Other*** |
| *Methods* | | | |
| **1** Grouping studies for synthesis | 1a) Provide a description of, and rationale for, the groups used in the synthesis (e.g., groupings of populations, interventions, outcomes, study design) | Methods 2.6 |  |
|  | 1b) Detail and provide rationale for any changes made subsequent to the protocol in the groups used in the synthesis | n/a |  |
| **2** Describe the standardised metric and transformation methods used | Describe the standardised metric for each outcome. Explain why the metric(s) was chosen, and describe any methods used to transform the intervention effects, as reported in the study, to the standardised metric, citing any methodological guidance consulted | n/a |  |
| **3** Describe the synthesis methods | Describe and justify the methods used to synthesise the effects for each outcome when it was not possible to undertake a meta-analysis of effect estimates | Methods 2.6 |  |
| **4** Criteria used to prioritise results for summary and synthesis | Where applicable, provide the criteria used, with supporting justification, to select the particular studies, or a particular study, for the main synthesis or to draw conclusions from the synthesis (e.g., based on study design, risk of bias assessments, directness in relation to the review question) | Methods 2.6 |  |
| **SWiM reporting item** | **Item description** | **Page in manuscript where item is reported** | **Other*** |
| **5** Investigation of heterogeneity in reported effects | State the method(s) used to examine heterogeneity in reported effects when it was not possible to undertake a meta-analysis of effect estimates and its extensions to investigate heterogeneity | Exploration of heterogeneity reported in 4.1 |  |
| **6** Certainty of evidence | Describe the methods used to assess certainty of the synthesis findings | Methods 2.4 |  |
| **7** Data presentation methods | Describe the graphical and tabular methods used to present the effects (e.g., tables, forest plots, harvest plots).  Specify key study characteristics (e.g., study design, risk of bias) used to order the studies, in the text and any tables or graphs, clearly referencing the studies included | Methods 2.6 |  |
| *Results* | | | |
| **8** Reporting results | For each comparison and outcome, provide a description of the synthesised findings, and the certainty of the findings. Describe the result in language that is consistent with the question the synthesis addresses, and indicate which studies contribute to the synthesis | Results 3.4 |  |
| *Discussion* |  |  |  |
| **9** Limitations of the synthesis | Report the limitations of the synthesis methods used and/or the groupings used in the synthesis, and how these affect the conclusions that can be drawn in relation to the original review question | Discussion 4.3 |  |

PRISMA=Preferred Reporting Items for Systematic Reviews and Meta-Analyses.

*If the information is not provided in the systematic review, give details of where this information is available (e.g., protocol, other published papers (provide citation details), or website (provide the URL)).

## 4 – Item 5, Data Extraction

| **Author,**  **Year** | **Sample Size, Age** | **Cohort / Population** | **Country** | **Exposure Measure**  **(SES)** | **Outcome Variable** | **Outcome Measure** | **Statistical Methods** | **Confounders**  **Adjusted for** |
| --- | --- | --- | --- | --- | --- | --- | --- | --- |
| **Trajectory Analysis** | | | | | | | | |
| Cadar, 2018 (13) | 2,032  Aged ≥65 | ELSA cohort | UK | **Child SES:** Highest level of education  **Adult SES:** Occupation, wealth  **Social mobility:** Combination of child SES and adult occupational class into three stable SES levels (low, medium, high), noting upwards or downwards trends | Dementia | Dementia was determined by doctor-diagnosis combined with a score above the threshold of 3.38 on the IQOCDE | Multivariable logistic regressions | Age, sex, marital status, long-standing limiting illness |
| Cha,  2021 (14) | 3,592  Aged ≥65 | HRS cohort | US | **Child SES:** Paternal and maternal education, and participant's education level **Adult SES:** Late-life wealth | Dementia | Langa-Weir classification approach. Immediate word recall (0-10), delayed word recall (0-10), serial subtraction of 7s (0–5), and backward counting from 20 (0–2), with a total score range of 0 to 27. Scoring <6 indicated dementia. Proxies interviewed if participant unable | Multivariate hazard models and Stochastic Population Analysis for Complex Events (SPACE) to simulate life paths for specific SES characteristics | Age, sex |
| Marden,  2017 (21) | 10,781  Aged ≥50, (mean 69) | HRS cohort | US | **Child SES:** Parental education, paternal occupation, and financial capital **Early adult SES:** Educational attainment as proxy for early adult SES **Late-life SES:** Self-reported household income in 2000, and current labour-force status (works full-time, part-time, or retired vs. unemployed, disabled, or not in labour force) | Memory Decline | Immediate and delayed recall of a 10-word list and the IQOCDE. Proxies interviewed if participant unable | Marginal mean regression models for repeated outcomes to model memory function longitudinally. Inverse probability weights accounted for differential attrition and selective survival | Birth year, race/ethnicity, sex, southern birthplace, marital status, age at first child's birth, smoking initiation age, childhood social capital, maternal investment, father presence/absence in childhood, urban/rural childhood residence, childhood and baseline self-rated health, smoking status, alcohol consumption, BMI, physical activity, diabetes, hypertension, depressive symptoms, heart disease/stroke diagnosis |
| Schrempft, 2023 (24) | 2,203  Mean ages: 72 (CoLaus\|PsyCoLaus) 75 (VLV) | 1,210 members of CoLaus cohort  993 members of VLV cohort | Switzerl-and | **Child SES:** Paternal occupation and highest parental education. In CoLaus, additional factors included family car, TV, dishwasher, telephone, home ownership, sufficient heat, cultural/social participation, holidays, employing a maid **Adult SES:** Highest education level, last known occupation, household gross monthly income **Social Mobility**: Categorised childhood paternal occupation and participant’s last known occupation (stable-low, downward, stable-mid, upward, stable-high) | Cognitive Decline | Verbal fluency: phonemic and semantic tasks (both cohorts). Processing Speed: Trail Making Test A (VLV), Stroop colour condition (CoLaus). Cognitive flexibility: Trail Making Test B (VLV), verbal fluency category switching (CoLaus). Inhibitory control: Stroop colour-word task (CoLaus). Verbal memory: the Wechsler Memory Scale (VLV), Grober and Buschke Double Memory Test (CoLaus). Subjective cognitive complaints: the Cognitive Complaint Questionnaire (CoLaus). Global cognition: the MMSE (both cohorts) and the CDR (CoLaus) | Linear regression and logistic regression. Longitudinal associations were examined using standardised change scores for continuous cognitive outcomes. Models controlled for covariates and baseline outcome levels | Age, sex, age squared (to account for non-linear effects of age on cognition), cardiovascular disease, diabetes, hypertension, smoking status, alcohol consumption, physical inactivity, comorbidities and depressive symptoms |
| Sindi,  2019 (25) | 1,409  Mean age 50 at baseline | CAIDE cohort | Finland | **Adult SES:** Self-rated satisfaction with financial situation at midlife (5-point Likert Scale), longest held occupation  **Social Mobility:** Participants' rated whether they had any change in financial situation at midlife that improved or worsened their financial situation compared to before (categories: better, same as before or worse than before) | Dementia | MMSE scores informed referral to clinical phase. This involved neurological, neuropsychological, cardiovascular exams, brain imaging, blood tests, cerebrospinal fluid analysis and ECG. A review board (physician, neuropsychologist, senior neurologist) reviewed all assessments. Dementia was diagnosed using DSM criteria (1994) and Alzheimer’s disease using NINCDS-ADRDA criteria | Logistic regression. Results correspond to the analysis of the pooled risk of having incident dementia (since previous time point) at first and second re-examination | Age, sex, education, annual baseline household income, Apolipoprotein ε4 allele status. Subsequent analyses also adjusted for cardio / cerebrovascular conditions and hopelessness (model 2), and type of occupation (model 3) |
| Staff,  2018 (26) | 388  Aged 62-77 | Sub-sample of Aberdeen 1936 birth cohort | UK | **Child SES:** Self-reported home conditions, paternal occupational social class at aged 11. Years in education before aged 25 **Adult SES:** Occupational Social Class in adulthood, residential deprivation applying the Scottish Index of Multiple Deprivation  **Social Mobility:** Relative standardised difference between child and adult SES | Memory Decline | Psychological tests administered by a psychologist following standard procedures. The Rey Auditory-Verbal Learning Test measured age-related memory decline | Multilevel linear modelling.  Model 1: Main effects of age, sex, practice, childhood ability, SES, and social mobility on late-life memory. Model 2 added interaction effects: Age × Sex, Age × child SES, and Age × social mobility.  Model 3 removed childhood ability from Model 2 to test changes in associations | Age, sex, practice effects, childhood ability (based on the Moray House Test scores) |
| Zeki Al  Hazzouri  2011 (27) | 1,789  Aged ≥60 | SALSA cohort, participants of Mexican origin | US | **Child SES:** Parental education and occupation, food deprivation growing up, childhood sibling mortality  **Early adult and midlife SES:** participants' educational attainment and lifetime occupation **Social Mobility:** Sum of dichotomised child, early adulthood, and midlife SES measures | Dementia | Modified MMSE, the Spanish and English Verbal Learning Test, and a memory word list recall test. Those scoring below the 20th percentile or with significant declines underwent further neuropsychological testing. Diagnoses were made using DSM-4 and NINCDS-ADRDA criteria, classifying participants as normal, cognitively impaired, or demented. Dementia cases underwent MRI and lab tests. Dementia and CIND were combined into one outcome: dementia/CIND | Cox Proportional Hazards Models. Bivariate associations between covariates and risk of dementia/CIND were analysed. Dementia/CIND cases were divided by person-years at risk within each SES trajectory to calculate incidence rates | Nativity (US-born or Mexican-born), past-month household income, fasting blood glucose levels, blood pressure, diabetes, hypertension, stroke event, height, weight, BMI, waist circumference, health insurance status, baseline smoking status, alcohol consumption |
| **Mediation Analysis** | | | | | | | | |
| Baranyi,  2023 (12) | 1,901  Aged ≥70 | Lothian Birth Cohort 1936 | UK | ‘Lifegrid’ questionnaire: retrospective residential history for each decade from birth to completion. Decade-specific neighbourhood social deprivation scores for Edinburgh were constructed using historical indices for 1941-1971 and the Carstairs index for 1981-2011. Neighbourhood social deprivation scores were linked to participants' residential history in 10-year time bands | Cognitive Decline | 10 cognitive tests grouped into three domains: Visuospatial Ability: Block Design and Matrix Reasoning and Spatial Span. Memory: Logical Memory, Verbal Paired Associates, and Backward Digit Span. Processing Speed: Digit Symbol Substitution, Symbol Search, and Experimental Tasks: Four-Choice Reaction Time, Inspection Time | Latent growth curve modelling within a structural equation modelling framework with FIML. A hierarchical ‘factor-of-curves’ approach calculated intercepts at age 70 and slopes of cognitive test scores between aged 70-82. Path models explored associations between life-course neighbourhood deprivation and late-life cognitive function, using two nested models | Age, sex, parental occupational social class, apolipoprotein ε4 allele status, childhood IQ, years spent in full-time education, adult occupational social class. Health indicators in sensitivity analysis: smoking status, BMI, history of self-reported medical diagnoses (cardiovascular disease, diabetes, hypertension, stroke) |
| Chiao,  2014 (15) | 2,944  Aged ≥60 | TLSA cohort | Taiwan | **Child SES:** Paternal education and occupation **Adult SES:** Participant’s education and primary lifetime occupation **Social Mobility:** Education and occupation measures combined to make a four category composite index: high SES (high for both), medium SES (low for one), low SES (low for both), or missing for both | Cognitive Decline | In-person interviews and five items from the Short Portable Mental Status Questionnaire, validated for Chinese equivalents of the MMSE and as a measure of cognitive functioning used in the TLSA | Mixed-effects models fitted three-parameter growth curves to repeated cognitive scores: intercept (baseline), slope (linear decline), and practice effect (step increase after first test). A sequential modelling strategy assessed cumulative SES disadvantage and cognitive changes. Model 1: child SES effects, Model 2: adult SES effects, Model 3: both child and adult SES effects | Age, sex, marital status, household income, self-rated health, physical disability, a history of cardiovascular disease, psychological distress, smoking, alcohol use, and life-related stressor and buffering variables (measures of social support, social participation and economic strain) |
| Deckers 2019 (16) | 6,346  Aged ≥50, (mean 64.9) | ELSA cohort | UK | **Child SES:** Highest education level achieved  **Adult SES:** Self-reported household wealth | Dementia | Combined algorithm of physician-diagnosed dementia or Alzheimer’s disease self-reported by the participant or their informant during the computer-assisted personal interview, or an average score ≥ 3.38 on the shortened IQOCDE | Structural equation modelling for continuous-time survival analysis (Cox proportional hazard regression) examined the associations between SES, LIBRA and time to dementia, resulting in hazard ratios and their 95% confidence intervals | Age, sex, wealth/education, clustering at household level and a weighted compound score of twelve modifiable risk and protective factors for dementia (LIBRA score) |
| Korhonen, 2023 (19) | 95,381  Aged 49-64 | 10% sample of 1950 Finnish population census linked to population and health registers | Finland | **Child SES:** Data from ages 0–15 in the Finnish population census including parents’ education, occupational social class, home ownership, geographical region of residence, household crowding, family structure and housing conditions **Adult SES:** Education, occupational social class, household disposable income, and main economic activity at age 50 (employed, unemployed, non-employed) | Dementia | Hospital, medication, and death registers covering the entire population. Dementia incidence was defined as the first entry in any of these registers | Discrete time survival model with logistic regression estimating odds ratios for dementia by childhood characteristics. Mediation through adult SES and cardiovascular health was analysed using the Karlson–Holm–Breen method. Total effect model included only childhood characteristics, while the direct effect model adjusted for adult SES and cardiovascular health. The proportion mediated was calculated as the indirect effect divided by the total effect. Cox proportional hazards models confirmed results | Age, sex, region of residence in 1950, calendar year (model 1), childhood characteristics and family type (model 2), marital status, alcohol-related diseases and accidental poisoning by alcohol, diabetes, dyslipidaemia and hypertension, having a CVD (ischaemic heart disease, cerebrovascular disease, heart failure, atrial fibrillation or peripheral arterial disease) |
| Krasanova, 2023 (20) | 1,746  Aged ≥65  Mean baseline age 80.4 | Rush Memory and Aging Project cohort | US | **Child SES:** Mean years of parental education, paternal `principal occupation, number of children in the family **Young-Adult SES:**  Years of education between ages 0-29  **Midlife SES:** Total family income at age 40 (including wages, salaries, social security or retirement benefits, help from relatives and rent from property)  **Late-life SES:**  Income at study enrolment aged 80 years on average measured in the same ways as at age 40 | Cognitive Decline | Nineteen cognitive tests were administered at baseline and annually at follow-up. Five cognitive domains were assessed: episodic memory, semantic memory, working memory, visuospatial ability, and perceptual speed. Global cognition was based on the average of test scores | Controlled direct effect of child SES on cognitive level and decline was calculated, fixing subsequent SES measures. Controlled direct effects for young-adulthood, midlife, and late-life SES on late-life cognitive function and decline were adjusted for time-dependent confounding. Marginal structural models with repeated outcome measures estimated causal effects with time-dependent confounders, using inverse probability weighting to adjust for confounders, survival, and missing data. Multiple imputation addressed selection bias | Child SES: Birth year, sex, race/ethnicity. Young adult SES: child SES, emotional neglect, family problems and family separation up to aged 18. Midlife: Young-adult SES and corresponding confounders. Late-life: Midlife SES, corresponding confounders, and baseline: age, marital status, BMI, smoking status, alcohol use, participation in social activities, physical activity, history of heart conditions, diabetes, hypertension, stroke and head injury |
| Oi,  2019 (22) | 9,449  Age range NR | HRS cohort | US | **Child SES:** Parental education, occupation of father/main breadwinner  **Adult SES:** Household income, wealth, cognitive demands of longest held occupation, educational attainment | Cognitive Decline | Modified version of the TICS | Latent growth curves of cognitive function were estimated within a structural equation framework. Estimations using FIML | Age at baseline, sex, race-ethnicity |
| Racine  Maurice,  2021 (23) | 519  Mean age 69.5 | LBC1936 cohort | UK | **Child SES:** Household crowding at aged 11, paternal occupation, each parents’ educational attainment  **Adult SES:** Participant’s occupational class | Cognitive Decline | MMSE at waves 1-3. Cognitive decline was determined by subtracting the greater of the first two MMSE scores obtained (wave 1 or 2) from wave 3 MMSE scores | Regression analysis. The study applied the critical period model by controlling for adult SES to examine the direct effect of child SES on cognitive decline. It also analysed adult SES as a mediator of the indirect relationship between child SES and cognitive decline, reflecting the pathway model | No adjustment for confounders mentioned |
| Zhang,  2008 (29) | 8,444  Aged 80- 105 | CLHLS cohort | China | **Child SES:** Place of birth, whether the respondent frequently went to bed hungry in childhood, and education **Adult SES:** Primary lifetime occupation before age 60 and current residence (urban vs. rural) | Incident cognitive Impairme-nt | The Chinese version of MMSE, testing orientation, calculation, recall, and language. An MMSE score of 18 was used as the cut-off for defining moderate-severe cognitive impairment. If scores fell below 18 during the 2-year follow up this was classed as onset of cognitive impairment | Multinomial logistic regression to model child SES association with cognitive impairment onset over two years (death and attrition as competing risks). A nested modelling strategy evaluated child and adult SES associations with cognitive impairment onset. A reduced model identified total effect of child SES on cognitive impairment odds, and an expanded model assessed if adding adult SES reduced this effect | Age, marital status, number and frequency of children visiting, involvement in leisure activities, disability in 1998 were all included in the incidence model |
| **Both Analyses** | | | | | | | | |
| Faul,  2021 (17) | 29,237  Aged ≥50  (HRS and ELSA) | 23,229 members of HRS cohort  6,008 members of ELSA cohort | US and UK | **Child SES:** Parental unemployment and occupation, experience of financial difficulty before aged 16. An index of child SES was made by dichotomising all childhood variables  **Adult SES:** Participant's education and baseline wealth  **Social Mobility:** Eight SES mobility trajectories (‘HHH’ = high child SES index, high education and high wealth) reflect respondent’s SES trajectory from childhood, through early adulthood, to mid- and later life. | Cognitive Decline | HRS: Episodic memory tasks assessing delayed recall  ELSA: Memory, processing speed, and executive functioning tests assessing immediate and delayed recall | Mixed effect models with random effects using FIML with an unstructured covariance matrix. A pooled analysis of HRS and ELSA data checked for cohort differences in cognitive performance, change in cognitive performance, and the effect of social mobility | Age, sex, race/ethnicity, marital/partner status, cohort and cognitive test practice effect |
| Karp,  2004 (18) | 931  Aged ≥75 | Members of Kungsholmen Project | Sweden | **Child SES:** Total years of formal education **Adult SES:** Longest held occupation **Social Mobility:** Education and occupation-based SES (at age 20, 40, and 60) to estimate individual mobility patterns: high education high SES, high education low SES, low education high SES, low education low SES | Dementia | DSM-3 revised edition criteria with a three-step procedure: 1) Physician examination, 2) Review by specialised clinician and second diagnosis, and 3) Third opinion in case of disagreement | Cox proportional hazards regression analyses | Age, gender, vascular diseases, alcohol data. Further analyses included cognitive status, social network and engagement in mental and physical activities, all at baseline |
| Zeng,  2022 (28) | 8,376  Aged ≥50 | HRS cohort | US | **Child SES:** Parental education and occupation, self-reported financial status (between age 0-16) **Adult SES:** Education, longest career occupation, household income, net worth **Social Mobility:** Summed SES conditions across life stages (stable high, downward, upward, stable low) | Cognitive Decline | Abbreviated version of the TICS, reporting global cognitive assessment, and memory-specific domain | Latent growth curve models. Analyses using FIML estimation with Huber-White adjustment | Age, sex, race, activities of daily living and instrumental activities of daily living, sum of chronic diseases scores, practice effect variables, survey design |

**Supplementary Material, Item 5** Characteristics of the included studies, stratified by analytic design. CAIDE: Cardiovascular Risk Factors, Aging, and Dementia, CIND: Cognitive Impairment Not Dementia, CLHLS: Chinese Longitudinal Healthy Longevity Survey, DSM: Diagnostic and Statistical Manual of Mental Disorders, ECG: Electrocardiogram, ELSA: English Longitudinal Survey of Ageing, FIML: Full Information Maximum Likelihood Estimation, HRS: Health and Retirement Study, IQOCDE: Informant Questionnaire on Cognitive Decline in the Elderly, LBC1936: Lothian Birth Cohort 1936, LIBRA score: LIfestyle for BRAin Health score, MMSE: Mini-Mental State Examination, MRI: Magnetic Resonance Imaging, NINCDS-ADRDA: National Institute of Neurological and Communicative Disorders and Stroke and the Alzheimer's Disease and Related Disorders Association, Age range NR: Age range not reported, SALSA: Sacramento Area Latino Study on Aging, SES: Socioeconomic Status, TICS: Telephone Interview of Cognitive Status, TLSA: Taiwan Longitudinal Study on Aging, VLV: Vivre/Leben/Vivere cohort.

## Item 6 – Risk of Bias Assessment

| **Author, Year** | **1** | **2** | **3** | **4** | **5** | **6** | **7** | **8** | **9** | **10** | **11** |  |
| --- | --- | --- | --- | --- | --- | --- | --- | --- | --- | --- | --- | --- |
| **Trajectory Analysis** | | | | | | | | | | | | |
| Cadar, 2018 (13) | Yes | Yes | Min C | Min C | Yes | U | Yes | Yes | No | U | Yes |  |
| Cha, 2021 (14) | Yes | Yes | Yes | Min C | Yes | Yes | Yes | Yes | No | No | Yes |  |
| Marden, 2017 (21) | Yes | Yes | Yes | Yes | Yes | / | Yes | Yes | Yes | Yes | Yes |  |
| Schrempft, 2023 (24) | Yes | Yes | Yes | Yes | Yes | / | Yes | Yes | Yes | No | Yes |  |
| Sindi, 2019 (25) | Yes | Yes | No | Min C | Yes | Yes | Yes | Yes | Yes | No | Yes |  |
| Staff, 2018 (26) | Yes | Yes | Yes | Min C | Yes | / | Yes | Yes | Yes | No | Yes |  |
| Zeki Al Hazzouri, 2011 (27) | Yes | Yes | Min C | Yes | Yes | Yes | Yes | Yes | Yes | Yes | Yes |  |
| **Mediation Analysis** | | | | | | | | | | | | |
| Baranyi, 2023 (12) | Yes | Yes | Yes | Yes | Yes | / | Yes | Yes | Yes | Yes | Yes |  |
| Chiao, 2014 (15) | Yes | Yes | Min C | Yes | Yes | / | Maj C | Yes | Yes | No | No |  |
| Deckers, 2019 (16) | Yes | Yes | Yes | Yes | Yes | Yes | Yes | Yes | Yes | Yes | Yes |  |
| Korhonen, 2023 (19) | Yes | Yes | Yes | Min C | Yes | Yes | Yes | Yes | Yes | Yes | Yes |  |
| Krasanova, 2023 (20) | Yes | Yes | Yes | Yes | Yes | / | Yes | Yes | Yes | Yes | Yes |  |
| Oi, 2019 (22) | Yes | Yes | Yes | Min C | Yes | / | Yes | Yes | No | Yes | Yes |  |
| Racine Maurice, 2021 (23) | Yes | Yes | Yes | Maj C | No | / | Yes | Yes | Yes | No | Yes |  |
| Zhang, 2008 (29) | Yes | Yes | No | Min C | Yes | / | Min C | Yes | Yes | Yes | Yes |  |
| **Both** | | | | | | | | | | | | |
| Faul, 2021 (17) | Yes | Yes | Yes | Min C | Yes | / | Yes | Yes | No | Yes | Yes |  |
| Karp, 2004 (18) | Yes | Yes | Min C | Yes | Yes | Yes | Yes | U | Yes | No | Yes |  |
| Zeng, 2022 (28) | Yes | Yes | Yes | Min C | Yes | / | Yes | Yes | Yes | Yes | Yes |  |

**Supplementary Material, Item 6** Risk of Bias Assessment for studies included in the systematic review using the JBI Appraisal Checklist (11). Maj C: Major Concern, Min C: Minor Concern, U: Unclear, /: Not applicable. To refine our assessment, we introduced the additional terms ‘minor’ and ‘major’ concerns. Specifically, minor concerns were marked for cases where ‘no’ was not applicable, but the criteria had not been met sufficiently to class as ‘yes’. Major concerns were used to highlight that the omission of a certain criteria was a particular weakness in the study and a major risk of bias to consider. Lastly, for studies measuring cognitive decline or function, criteria 6 of the JBI checklist was marked as ‘not applicable’ as participants cannot be free of cognitive function or cognitive decline at baseline. See supplementary materials document for the complete JBI critical appraisal checklist for cohort studies.

## Item 7 - Search strategy update – 20/08/25

**Ovid MEDLINE(R) and Epub Ahead of Print, In-Process, In-Data-Review & Other Non-Indexed Citations, Daily and Versions <1946 to August 19, 2025>**

1 (Social class or Social position or Social standing or Social status or socioeconomic or Socio*economic status or Socio*economic class or Socio*economic position or Socio*economic standing or education or occupation or income or poverty or wealth).ti,ab,kw,kf. or exp Social Class/ or Socioeconomic Factors/ or Sociodemographic Factors/ or income/ or exp career mobility/ or economic status/ or educational status/ 1229465

2 (Dementia or Alzheimer* Disease or Vascular dementia or Lewy Bod* or mixed dementia or Cognitive decline or Cognitive reserve or Cognitive resilience or Brain reserve or Cognitive Impairment).ti,ab,kw,kf. or Dementia/ or Alzheimer Disease/ or Dementia, Vascular/ or Lewy Bod*/ or Mixed Dementias/ 408538

3 (longitudinal stud* or longitudinal survey* or longitudinal analys* or prospective stud* or prospective cohort* or follow up or followup* or follow-up stud* or cohort stud* or mediation analys* or association analys* or attenuat* or inciden* or prevalen* or lifecourse or life course or life-course).ti,ab,kw,kf. or longitudinal studies/ or prospective studies/ or Cohort Studies/ 4874408

4 (trajectory or life course or lifecourse or mediat* or attenuat* or trend* or change or childhood or adulthood).ti,ab,kw,kf. or Social Mobility/ or exp career mobility/ 4458741

5 1 and 2 and 3 and 4 3124

6 limit 5 to dt=20240222-20250819 494

**Embase <1996 to 2025 Week 33>**

1 (Social class or Social position or Social standing or Social status or socioeconomic or Socio*economic status or Socio*economic class or Socio*economic position or Socio*economic standing or education or occupation or income or poverty or wealth).ti,ab. 1177694

2 exp *social class/ or exp *socioeconomics/ or exp *sociodemographics/ or exp *income/ or exp *career mobility/ or exp *economic status/ or exp *educational status/ 255838

3 1 or 2 1350538

4 (Dementia or Alzheimer* Disease or Vascular dementia or Lewy Bod* or mixed dementia or Cognitive decline or Cognitive reserve or Cognitive resilience or Brain reserve or Cognitive Impairment).ti,ab. 483642

5 exp *dementia/ or exp *Alzheimer disease/ or exp *multiinfarct dementia/ or exp *Lewy body/ or exp *mixed dementia/ 267545

6 4 or 5 536288

7 (longitudinal stud* or longitudinal survey* or longitudinal analys* or prospective stud* or prospective cohort* or follow up or followup* or follow-up stud* or cohort stud* or mediation analys* or association analys* or attenuat* or inciden* or prevalen* or lifecourse or life course or life-course).ti,ab. 5790794

8 exp *longitudinal study/ or exp *prospective study/ or exp *cohort analysis/ 95290

9 7 or 8 5806547

10 (trajectory or life course or lifecourse or mediat* or attenuat* or trend* or change or childhood or adulthood).ti,ab. 5025268

11 exp *social mobility/ or exp *career mobility/ 4082

12 10 or 11 5029016

13 3 and 6 and 9 and 12 4324

14 limit 13 to dd=20240221-20250820 543

**# Web of Science Search Strategy (v0.1)**

# Database: Web of Science Core Collection

# Entitlements:

- WOS.IC: 1993 to 2025

- WOS.CCR: 1985 to 2025

- WOS.SCI: 1900 to 2025

- WOS.AHCI: 1975 to 2025

- WOS.BHCI: 2008 to 2025

- WOS.BSCI: 2008 to 2025

- WOS.ESCI: 2020 to 2025

- WOS.ISTP: 1990 to 2025

- WOS.SSCI: 1956 to 2025

- WOS.ISSHP: 1990 to 2025

# Searches:

1: ( TI= ( ( "LONGITUDINAL STUD*" OR "LONGITUDINAL SURVEY*" OR "LONGITUDINAL ANALYS*" OR "PROSPECTIVE STUD*" OR "PROSPECTIVE COHORT*" OR "FOLLOW UP" OR FOLLOWUP* OR "FOLLOW-UP STUD*" OR "COHORT STUD*" OR "MEDIATION ANALYS*" OR "ASSOCIATION ANALYS*" OR ATTENUAT* OR INCIDEN* OR PREVALEN* OR LIFECOURSE OR "LIFE COURSE" OR "LIFE-COURSE" ) ) ) OR ( AB= ( ( "LONGITUDINAL STUD*" OR "LONGITUDINAL SURVEY*" OR "LONGITUDINAL ANALYS*" OR "PROSPECTIVE STUD*" OR "PROSPECTIVE COHORT*" OR "FOLLOW UP" OR FOLLOWUP* OR "FOLLOW-UP STUD*" OR "COHORT STUD*" OR "MEDIATION ANALYS*" OR "ASSOCIATION ANALYS*" OR ATTENUAT* OR INCIDEN* OR PREVALEN* OR LIFECOURSE OR "LIFE COURSE" OR "LIFE-COURSE" ) ) ) Date Run: Wed Aug 20 2025 10:32:42 GMT+0100 (British Summer Time) Results: 4875815

2: ( TI= ( ( DEMENTIA OR "ALZHEIMER* DISEASE" OR "VASCULAR DEMENTIA" OR "LEWY BOD*" OR "MIXED DEMENTIA" OR "COGNITIVE DECLINE" OR "COGNITIVE RESERVE" OR "COGNITIVE RESILIENCE" OR "BRAIN RESERVE" OR "COGNITIVE IMPAIRMENT" ) ) ) OR ( AB= ( ( DEMENTIA OR "ALZHEIMER* DISEASE" OR "VASCULAR DEMENTIA" OR "LEWY BOD*" OR "MIXED DEMENTIA" OR "COGNITIVE DECLINE" OR "COGNITIVE RESERVE" OR "COGNITIVE RESILIENCE" OR "BRAIN RESERVE" OR "COGNITIVE IMPAIRMENT" ) ) ) Date Run: Wed Aug 20 2025 10:33:31 GMT+0100 (British Summer Time) Results: 302006

3: ( TI= ( ( "SOCIAL CLASS" OR "SOCIAL POSITION" OR "SOCIAL STANDING" OR "SOCIAL STATUS" OR SOCIOECONOMIC OR "SOCIO?ECONOMIC STATUS" OR "SOCIO?ECONOMIC CLASS" OR "SOCIO?ECONOMIC POSITION" OR "SOCIO?ECONOMIC STANDING" OR EDUCATION OR OCCUPATION OR INCOME OR POVERTY OR WEALTH ) ) ) OR ( AB= ( ( "SOCIAL CLASS" OR "SOCIAL POSITION" OR "SOCIAL STANDING" OR "SOCIAL STATUS" OR SOCIOECONOMIC OR "SOCIO?ECONOMIC STATUS" OR "SOCIO?ECONOMIC CLASS" OR "SOCIO?ECONOMIC POSITION" OR "SOCIO?ECONOMIC STANDING" OR EDUCATION OR OCCUPATION OR INCOME OR POVERTY OR WEALTH ) ) ) Date Run: Wed Aug 20 2025 10:33:48 GMT+0100 (British Summer Time) Results: 2012793

4: ( TI= ( ( TRAJECTORY OR "LIFE COURSE" OR LIFECOURSE OR MEDIAT* OR ATTENUAT* OR TREND* OR CHANGE OR CHILDHOOD OR ADULTHOOD ) ) ) OR ( AB= ( ( TRAJECTORY OR "LIFE COURSE" OR LIFECOURSE OR MEDIAT* OR ATTENUAT* OR TREND* OR CHANGE OR CHILDHOOD OR ADULTHOOD ) ) ) Date Run: Wed Aug 20 2025 10:34:16 GMT+0100 (British Summer Time) Results: 11612781

5: #4 AND #3 AND #2 AND #1 Date Run: Wed Aug 20 2025 10:34:31 GMT+0100 (British Summer Time) Results: 2994

6: #1 AND #2 AND #3 AND #4 Timespan: 2024-02-20 to 2025-08-20 Date Run: Wed Aug 20 2025 10:36:55 GMT+0100 (British Summer Time) Results: 521

**Scopus**

( TITLE-ABS ( ( "LONGITUDINAL STUD*" OR "LONGITUDINAL SURVEY*" OR "LONGITUDINAL ANALYS*" OR "PROSPECTIVE STUD*" OR "PROSPECTIVE COHORT*" OR "FOLLOW UP" OR FOLLOWUP* OR "FOLLOW-UP STUD*" OR "COHORT STUD*" OR "MEDIATION ANALYS*" OR "ASSOCIATION ANALYS*" OR ATTENUAT* OR INCIDEN* OR PREVALEN* OR LIFECOURSE OR "LIFE COURSE" OR "LIFE-COURSE" ) ) ) AND ( TITLE-ABS ( ( DEMENTIA OR "ALZHEIMER* DISEASE" OR "VASCULAR DEMENTIA" OR "LEWY BOD*" OR "MIXED DEMENTIA" OR "COGNITIVE DECLINE" OR "COGNITIVE RESERVE" OR "COGNITIVE RESILIENCE" OR "BRAIN RESERVE" OR "COGNITIVE IMPAIRMENT" ) ) ) AND ( TITLE-ABS ( ( "SOCIAL CLASS" OR "SOCIAL POSITION" OR "SOCIAL STANDING" OR "SOCIAL STATUS" OR SOCIOECONOMIC OR "SOCIO?ECONOMIC STATUS" OR "SOCIO?ECONOMIC CLASS" OR "SOCIO?ECONOMIC POSITION" OR "SOCIO?ECONOMIC STANDING" OR EDUCATION OR OCCUPATION OR INCOME OR POVERTY OR WEALTH ) ) ) AND ( TITLE-ABS ( ( TRAJECTORY OR "LIFE COURSE" OR LIFECOURSE OR MEDIAT* OR ATTENUAT* OR TREND* OR CHANGE OR CHILDHOOD OR ADULTHOOD ) ) ) AND PUBYEAR > 2023 AND PUBYEAR < 2026

n=632

**CINAHL**

| **#** | **Query** | **Limiters/Expanders** | **Last Run Via** | **Results** |
| --- | --- | --- | --- | --- |
| S1 | TI ( (Social class or Social position or Social standing or Social status or socioeconomic or Socio*economic status or Socio*economic class or Socio*economic position or Socio*economic standing or education or occupation or income or poverty or wealth) ) OR AB ( (Social class or Social position or Social standing or Social status or socioeconomic or Socio*economic status or Socio*economic class or Socio*economic position or Socio*economic standing or education or occupation or income or poverty or wealth)) | Expanders - Apply equivalent subjects  Search modes - Boolean/Phrase | Interface - EBSCOhost Research Databases  Search Screen - Advanced Search  Database - CINAHL | 484,933 |
| S2 | (MH "Social Class+") OR (MH "Social Status") OR (MH "Socioeconomic Factors+") OR (MH "Sociodemographic Factors") OR (MH "Income+") OR (MH "Career Mobility+") OR (MH "Economic Status") OR (MH "Educational Status") | Expanders - Apply equivalent subjects  Search modes - Boolean/Phrase | Interface - EBSCOhost Research Databases  Search Screen - Advanced Search  Database - CINAHL | 452,362 |
| S3 | S1 OR S2 | Expanders - Apply equivalent subjects  Search modes - Boolean/Phrase | Interface - EBSCOhost Research Databases  Search Screen - Advanced Search  Database - CINAHL | 811,519 |
| S4 | TI ( (Dementia or Alzheimer* Disease or Vascular dementia or Lewy Bod* or mixed dementia or Cognitive decline or Cognitive reserve or Cognitive resilience or Brain reserve or Cognitive Impairment) ) OR AB ( (Dementia or Alzheimer* Disease or Vascular dementia or Lewy Bod* or mixed dementia or Cognitive decline or Cognitive reserve or Cognitive resilience or Brain reserve or Cognitive Impairment) ) | Expanders - Apply equivalent subjects  Search modes - Boolean/Phrase | Interface - EBSCOhost Research Databases  Search Screen - Advanced Search  Database - CINAHL | 122,644 |
| S5 | (MH "Dementia+") OR (MH "Dementia, Vascular+") OR (MH "Lewy Body Disease") OR (MH "Alzheimer's Disease") OR (MH "Mixed Dementias") | Expanders - Apply equivalent subjects  Search modes - Boolean/Phrase | Interface - EBSCOhost Research Databases  Search Screen - Advanced Search  Database - CINAHL | 89,323 |
| S6 | S4 OR S5 | Expanders - Apply equivalent subjects  Search modes - Boolean/Phrase | Interface - EBSCOhost Research Databases  Search Screen - Advanced Search  Database - CINAHL | 145,216 |
| S7 | TI ( (longitudinal stud* or longitudinal survey* or longitudinal analys* or prospective stud* or prospective cohort* or follow up or followup* or follow-up stud* or cohort stud* or mediation analys* or association analys* or attenuat* or inciden* or prevalen* or lifecourse or life course or life-course) ) OR AB ( (longitudinal stud* or longitudinal survey* or longitudinal analys* or prospective stud* or prospective cohort* or follow up or followup* or follow-up stud* or cohort stud* or mediation analys* or association analys* or attenuat* or inciden* or prevalen* or lifecourse or life course or life-course) ) | Expanders - Apply equivalent subjects  Search modes - Boolean/Phrase | Interface - EBSCOhost Research Databases  Search Screen - Advanced Search  Database - CINAHL | 1,048,933 |
| S8 | (MH "Prospective Studies+") | Expanders - Apply equivalent subjects  Search modes - Boolean/Phrase | Interface - EBSCOhost Research Databases  Search Screen - Advanced Search  Database - CINAHL | 551,215 |
| S9 | S7 OR S8 | Expanders - Apply equivalent subjects  Search modes - Boolean/Phrase | Interface - EBSCOhost Research Databases  Search Screen - Advanced Search  Database - CINAHL | 1,317,333 |
| S10 | TI ( (trajectory or life course or lifecourse or mediat* or attenuat* or trend* or change or childhood or adulthood) ) OR AB ( (trajectory or life course or lifecourse or mediat* or attenuat* or trend* or change or childhood or adulthood) ) | Expanders - Apply equivalent subjects  Search modes - Boolean/Phrase | Interface - EBSCOhost Research Databases  Search Screen - Advanced Search  Database - CINAHL | 1,012,675 |
| S11 | (MH "Social Mobility") OR (MH "Career Mobility+") | Expanders - Apply equivalent subjects  Search modes - Boolean/Phrase | Interface - EBSCOhost Research Databases  Search Screen - Advanced Search  Database - CINAHL | 8,926 |
| S12 | S10 OR S11 | Expanders - Apply equivalent subjects  Search modes - Boolean/Phrase | Interface - EBSCOhost Research Databases  Search Screen - Advanced Search  Database - CINAHL | 1,020,615 |
| S13 | S3 AND S6 AND S9 AND S12 | Expanders - Apply equivalent subjects  Search modes - Boolean/Phrase | Interface - EBSCOhost Research Databases  Search Screen - Advanced Search  Database - CINAHL | 2,027  (217 when date limits applied) |

**British Education Index**

| **#** | **Query** | **Limiters/Expanders** | **Last Run Via** | **Results** |
| --- | --- | --- | --- | --- |
| S5 | S1 AND S2 AND S3 AND S4 | Expanders - Apply equivalent subjects Search modes - Boolean/Phrase | Interface - EBSCOhost Research Databases Search Screen - Basic Search Database - British Education Index | 3  (0 after date filters applied) |
| S4 | TI ( (trajectory or life course or lifecourse or mediat* or attenuat* or trend* or change or childhood or adulthood) ) OR AB ( (trajectory or life course or lifecourse or mediat* or attenuat* or trend* or change or childhood or adulthood) ) | Expanders - Apply equivalent subjects Search modes - Boolean/Phrase | Interface - EBSCOhost Research Databases Search Screen - Basic Search Database - British Education Index | 40,992 |
| S3 | TI ( (longitudinal stud* or longitudinal survey* or longitudinal analys* or prospective stud* or prospective cohort* or follow up or followup* or follow-up stud* or cohort stud* or mediation analys* or association analys* or attenuat* or inciden* or prevalen* or lifecourse or life course or life-course) ) OR AB ( (longitudinal stud* or longitudinal survey* or longitudinal analys* or prospective stud* or prospective cohort* or follow up or followup* or follow-up stud* or cohort stud* or mediation analys* or association analys* or attenuat* or inciden* or prevalen* or lifecourse or life course or life-course) ) | Expanders - Apply equivalent subjects Search modes - Boolean/Phrase | Interface - EBSCOhost Research Databases Search Screen - Basic Search Database - British Education Index | 11,917 |
| S2 | TI ( (Dementia or Alzheimer* Disease or Vascular dementia or Lewy Bod* or mixed dementia or Cognitive decline or Cognitive reserve or Cognitive resilience or Brain reserve or Cognitive Impairment) ) OR AB ( (Dementia or Alzheimer* Disease or Vascular dementia or Lewy Bod* or mixed dementia or Cognitive decline or Cognitive reserve or Cognitive resilience or Brain reserve or Cognitive Impairment) ) | Expanders - Apply equivalent subjects Search modes - Boolean/Phrase | Interface - EBSCOhost Research Databases Search Screen - Basic Search Database - British Education Index | 455 |
| S1 | TI ( (Social class or Social position or Social standing or Social status or socioeconomic or Socio*economic status or Socio*economic class or Socio*economic position or Socio*economic standing or education or occupation or income or poverty or wealth) ) OR AB ( (Social class or Social position or Social standing or Social status or socioeconomic or Socio*economic status or Socio*economic class or Socio*economic position or Socio*economic standing or education or occupation or income or poverty or wealth)) | Expanders - Apply equivalent subjects Search modes - Boolean/Phrase | Interface - EBSCOhost Research Databases Search Screen - Basic Search Database - British Education Index | 120,255 |

**PsychInfo**

| **#** | **Query** | **Limiters/Expanders** | **Last Run Via** | **Results** |
| --- | --- | --- | --- | --- |
| S13 | S3 AND S6 AND S9 AND S12 | Expanders - Apply equivalent subjects Search modes - Boolean/Phrase | Interface - EBSCOhost Research Databases Search Screen - Advanced Search Database - APA PsycInfo | 2,233 (196 when date filter applied) |
| S12 | S10 OR S11 | Expanders - Apply equivalent subjects Search modes - Boolean/Phrase | Interface - EBSCOhost Research Databases Search Screen - Advanced Search Database - APA PsycInfo | 1,269,566 |
| S11 | DE "Social Mobility" | Expanders - Apply equivalent subjects Search modes - Boolean/Phrase | Interface - EBSCOhost Research Databases Search Screen - Advanced Search Database - APA PsycInfo | 2,308 |
| S10 | TI ( (trajectory or life course or lifecourse or mediat* or attenuat* or trend* or change or childhood or adulthood) ) OR AB ( (trajectory or life course or lifecourse or mediat* or attenuat* or trend* or change or childhood or adulthood) ) | Expanders - Apply equivalent subjects Search modes - Boolean/Phrase | Interface - EBSCOhost Research Databases Search Screen - Advanced Search Database - APA PsycInfo | 1,268,038 |
| S9 | S7 OR S8 | Expanders - Apply equivalent subjects Search modes - Boolean/Phrase | Interface - EBSCOhost Research Databases Search Screen - Advanced Search Database - APA PsycInfo | 679,328 |
| S8 | ((DE "Longitudinal Studies" OR DE "Prospective Studies")) OR (DE "Cohort Analysis") | Expanders - Apply equivalent subjects Search modes - Boolean/Phrase | Interface - EBSCOhost Research Databases Search Screen - Advanced Search Database - APA PsycInfo | 90.689 |
| S7 | TI ( (longitudinal stud* or longitudinal survey* or longitudinal analys* or prospective stud* or prospective cohort* or follow up or followup* or follow-up stud* or cohort stud* or mediation analys* or association analys* or attenuat* or inciden* or prevalen* or lifecourse or life course or life-course) ) OR AB ( (longitudinal stud* or longitudinal survey* or longitudinal analys* or prospective stud* or prospective cohort* or follow up or followup* or follow-up stud* or cohort stud* or mediation analys* or association analys* or attenuat* or inciden* or prevalen* or lifecourse or life course or life-course) ) | Expanders - Apply equivalent subjects Search modes - Boolean/Phrase | Interface - EBSCOhost Research Databases Search Screen - Advanced Search Database - APA PsycInfo | 646,383 |
| S6 | S4 OR S5 | Expanders - Apply equivalent subjects Search modes - Boolean/Phrase | Interface - EBSCOhost Research Databases Search Screen - Advanced Search Database - APA PsycInfo | 175,138 |
| S5 | DE "Dementia" OR DE "AIDS Dementia Complex" OR DE "Alzheimer's Disease" OR DE "Dementia with Lewy Bodies" OR DE "Frontotemporal Lobar Degeneration" OR DE "Presenile Dementia" OR DE "Pseudodementia" OR DE "Senile Dementia" OR DE "Vascular Dementia" | Expanders - Apply equivalent subjects Search modes - Boolean/Phrase | Interface - EBSCOhost Research Databases Search Screen - Advanced Search Database - APA PsycInfo | 105,270 |
| S4 | TI ( (Dementia or Alzheimer* Disease or Vascular dementia or Lewy Bod* or mixed dementia or Cognitive decline or Cognitive reserve or Cognitive resilience or Brain reserve or Cognitive Impairment) ) OR AB ( (Dementia or Alzheimer* Disease or Vascular dementia or Lewy Bod* or mixed dementia or Cognitive decline or Cognitive reserve or Cognitive resilience or Brain reserve or Cognitive Impairment) ) | Expanders - Apply equivalent subjects Search modes - Boolean/Phrase | Interface - EBSCOhost Research Databases Search Screen - Advanced Search Database - APA PsycInfo | 169,694 |
| S3 | S1 OR S2 | Expanders - Apply equivalent subjects Search modes - Boolean/Phrase | Interface - EBSCOhost Research Databases Search Screen - Basic Search Database - APA PsycInfo | 677,968 |
| S2 | ((DE "Social Class" OR DE "Disadvantaged" OR DE "Lower Socioeconomic Status" OR DE "Middle Socioeconomic Status" OR DE "Upper Socioeconomic Status") OR (DE "Socioeconomic Factors" OR DE "Economic Disadvantage" OR DE "Economic Resources" OR DE "Employment Status" OR DE "Income Level" OR DE "Social Class" OR DE "Social Disadvantage" OR DE "Socioeconomic Disparities" OR DE "Socioeconomic Status")) OR (DE "Income Level" OR DE "Lower Income Level" OR DE "Middle Income Level" OR DE "Upper Income Level") | Expanders - Apply equivalent subjects Search modes - Boolean/Phrase | Interface - EBSCOhost Research Databases Search Screen - Advanced Search Database - APA PsycInfo | 127,475 |
| S1 | TI ( (Social class or Social position or Social standing or Social status or socioeconomic or Socio*economic status or Socio*economic class or Socio*economic position or Socio*economic standing or education or occupation or income or poverty or wealth) ) OR AB ( (Social class or Social position or Social standing or Social status or socioeconomic or Socio*economic status or Socio*economic class or Socio*economic position or Socio*economic standing or education or occupation or income or poverty or wealth)) | Expanders - Apply equivalent subjects Search modes - Boolean/Phrase | Interface - EBSCOhost Research Databases Search Screen - Advanced Search Database - APA PsycInfo | 624,167 |
